# Resveratrol Does Not Influence Metabolic Risk Markers Related to Cardiovascular Health in Overweight and Slightly Obese Subjects: A Randomized, Placebo-Controlled Crossover Trial

**DOI:** 10.1371/journal.pone.0118393

**Published:** 2015-03-19

**Authors:** Sanne M. van der Made, Jogchum Plat, Ronald P. Mensink

**Affiliations:** 1 Department of Human Biology, NUTRIM School for Nutrition, Toxicology and Metabolism, Maastricht University Medical Center, Maastricht,The Netherlands; 2 Top Institute of Food and Nutrition (TIFN), Wageningen, The Netherlands; University of Milan, ITALY

## Abstract

**Background:**

*In vitro* and animal studies have shown positive effects of resveratrol on lipid and lipoprotein metabolism, but human studies specifically designed to examine these effects are lacking.

**Objective:**

The primary outcome parameter of this study in overweight and slightly obese subjects was the effect of resveratrol on apoA-I concentrations. Secondary outcome parameters were effects on other markers of lipid and lipoprotein metabolism, glucose metabolism, and markers for inflammation and endothelial function.

**Design:**

This randomized, placebo-controlled crossover study was conducted in 45 overweight and slightly obese men (n = 25) and women (n = 20) with a mean age of 61 ± 7 years. Subjects received in random order resveratrol (150 mg per day) or placebo capsules for 4 weeks, separated by a 4-week wash-out period. Fasting blood samples were collected at baseline and at the end of each intervention period.

**Results:**

Compliance was excellent as indicated by capsule count and changes in resveratrol and dihydroresveratrol concentrations. No difference between resveratrol and placebo was found in any of the fasting serum or plasma metabolic risk markers (mean ± SD for differences between day 28 values of resveratrol vs. placebo: apoA-I; 0.00 ± 0.12 g/L (P = 0.791), apoB100; -0.01 ± 0.11 g/L (P = 0.545), HDL cholesterol; 0.00 ± 0.09 mmol/L (P = 0.721), LDL cholesterol -0.03 ± 0.57 mmol/L (P = 0.718), triacylglycerol; 0.10 ± 0.54 mmol/L (P = 0.687), glucose; -0.08 ± 0.28 mmol/L (P = 0.064), insulin; -0.3 ± 2.5 mU/L (P = 0.516)). Also, no effects on plasma markers for inflammation and endothelial function were observed. No adverse events related to resveratrol intake were observed.

**Conclusion:**

150 mg of daily resveratrol intake for 4 weeks does not change metabolic risk markers related to cardiovascular health in overweight and slightly obese men and women. Effects on glucose metabolism warrant further study.

**Trial Registration:**

ClinicalTrials.gov NCT01364961

## Introduction

The many approaches that exist to lower cardiovascular risk are mainly aimed at lowering LDL cholesterol concentrations. Based on epidemiological studies [1–4], increasing HDL cholesterol concentrations may also reduce cardiovascular risk, but this concept has been challenged by intervention studies [5–7]. However, a recent meta-analysis has suggested that increasing apolipoprotein A-I (apoA-I) concentrations is a more important target to reduce the risk of major cardiovascular events than increasing HDL cholesterol concentrations [8]. ApoA-I is the major protein constituent of the HDL particle, which plays a crucial role in many of the favorable effects attributed to HDL particles, including—next to their fundamental role in reverse cholesterol transport—anti-inflammatory, anti-thrombotic and anti-oxidative effects [9,10].

Epidemiological studies have shown a positive association between higher polyphenol intakes and reduced cardiovascular risk [11,12]. This association may be due to increased intakes of *trans-*resveratrol (3,5,4’-trihydroxystilbene), a polyphenol found in the skin of black grapes. Resveratrol may influence numerous mechanisms, which act on the progression as well as on the regression of atherosclerosis [13]. In fact, *in vitro* and animal studies have shown many positive effects of resveratrol on genes and proteins involved in lipid and glucose metabolism, including increases in the activities of peroxisome proliferator-activated receptor α (PPARα) and PPARγ [14,15].

While the results from *in vitro* and animal studies are promising, and an HDL cholesterol elevating effect of berries [16] and red wine [17], both of which contain resveratrol, has been reported, human studies with the primary aim to examine the effects of resveratrol on lipid and lipoprotein metabolism are scarce. Furthermore, human data onto the effect of resveratrol on markers for metabolic risk is limited and results are conflicting [18–20]. Therefore, the primary aim of the present study was to evaluate the effect of 4 wk resveratrol supplementation (150 mg/day, 99% pure *trans*-resveratrol [resVida provided by DSM Nutritional Products Ltd. (Kaiseraugst, Switzerland]) on apoA-I concentrations in overweight and slightly obese subjects with low HDL cholesterol concentrations. Secondary aims were to assess effects on other markers of lipid and lipoprotein metabolism, glucose metabolism, and markers for inflammation and endothelial function.

## Subjects and Methods

### Subjects

The protocol for this trial and supporting CONSORT checklist are available as supporting information; see S1 CONSORT Checklist and S1 Protocol.

Subjects were recruited via posters in university and hospital buildings and by advertisements in local newspapers. In addition, men and women who had participated in earlier studies at the Department of Human Biology (Maastricht University) were approached. Subjects came to university for two screening visits with an interval of ≥ 3 days. Fasting blood was sampled on both occasions for lipoprotein and glucose analyses. Also, height, weight and blood pressure were determined. Furthermore, subjects were asked to complete a general and medical questionnaire. Inclusion criteria were as follows: age between 45 and 70 y; mean high-density lipoprotein cholesterol concentration < 1.21 mmol/L for men and < 1.53 mmol/L for women, in order to include subjects with HDL cholesterol concentrations below mean values found in the PROCAM study [2]; mean serum total cholesterol concentration < 8.0 mmol/L; plasma glucose < 7.0 mmol/L; body mass index between 25–35 kg/m^2^; non-smoker; willing to abstain from resveratrol-rich products two weeks prior to the study and during the study; stable body weight (weight gain or loss < 3 kg in three months prior to screening visit); no indication for treatment with cholesterol-lowering drugs according to Dutch Cholesterol Consensus; no use of medication or a prescribed diet known to affect serum lipid or glucose metabolism; no active cardiovascular disease or recent (< 6 months) event such as acute myocardial infarction or cerebrovascular accident; willing to stop consumption of vitamin supplements, fish oil capsules or products rich in plant stanol or sterol esters two weeks prior to and during the study; self-reported high alcohol consumption (> 21 units / week for men or > 14 units / week for women); no abuse of drugs; no pregnant or breastfeeding women; no participation in another biomedical study within 1 month prior to the first screening visit; not having donated blood (as blood donor) within 1 month prior to the first screening visit, planning to donate blood during the study or within one month after finishing the study. Finally, subjects who were impossible or difficult to puncture as evidenced during the screening visits could not participate. Participants were informed about the aim of the study and nature and risk of the experimental procedures before their written informed consent was obtained. This study was conducted according to the guidelines laid down in the Declaration of Helsinki, approved by the Ethics Committee of the Maastricht University Medical Centre, and registered on 17 January 2011 at ClinicalTrials.gov as NCT01364961.

### Study design and products

The study had a double-blind, randomized, placebo-controlled, cross-over design with two 4-wk intervention periods separated by a 4-wk wash-out period. Subjects were allocated to start with either resveratrol or placebo capsules according to a pre-established computer generated randomization scheme, and were asked to take two capsules daily during lunch and dinner. Resveratrol (150 mg/day; resVida, 99.9% *trans*-resveratrol) and placebo capsules were kindly provided by DSM Nutritional Products Ltd. (Kaiseraugst, Switzerland). Capsules were provided in weekly containers, coded with either ‘A’ or ‘B’ to blind subjects and investigators. Four containers were provided to the volunteers at the start of each intervention periods and subjects were asked to return all containers including any unused capsules, which were counted as a measure of compliance. Two weeks prior to the study and throughout the total study period, subjects were asked to abstain from resveratrol-containing products (such as grapes, wine, berries, peanuts, peanut butter, soy and soy products and pomegranate). At the end of both experimental periods, subjects completed a food-frequency questionnaire (FFQ) in which food intake from the previous 4 wk was recorded. A dietitian immediately checked the FFQs and calculated energy and nutrient intakes of the subjects using the Dutch food-composition tables. Subjects were asked to record any signs of illnesses, use of medication or alcohol consumption in a study diary.

### Blood sampling

Fasting blood samples were taken by venipuncture at the start and at day 25 of each intervention period. At day 28 blood was sampled through an intravenous catheter using a Vacutainer system (Becton, Dickinson and Company, Franklin Lanes, NY, USA). On days preceding blood drawings, subjects were asked to avoid the intake of drinks containing alcohol or caffeine, and not to take part in any strenuous activity. Blood samples were taken from a forearm vein after an overnight fast (no food or drink after 8 PM, except for water), by the same person, and at the same location. Serum separator tubes (Becton, Dickinson and Company, Franklin Lanes, NY, USA) were used for the analysis of lipids and lipoproteins, insulin and hsCRP. Serum tubes were kept at room temperature for at least 30 minutes after sampling to allow clotting. Serum was obtained by centrifugation at 1300 × *g* for 15 minutes at 21°C. EDTA-coated tubes (Becton, Dickinson and Company, Franklin Lanes, NY, USA) were used for measurement of inflammatory markers, resveratrol and dihydroresveratrol, and clinical chemistry measurements. Sodium fluoride (NaF) tubes (Becton, Dickinson and Company, Franklin Lanes, NY, USA) were used for analysis of plasma glucose. After blood drawing, NaF and EDTA-coated tubes were kept on ice and centrifuged within 30 minutes. Plasma was obtained by centrifugation at 1300 × *g* for 15 minutes at 4°C. Serum and plasma samples were portioned into aliquots, snap-frozen in liquid N_2_, and stored at -80°C. Hemostatic and hematologic measurements were performed in whole blood from citrate-filled and EDTA-coated tubes respectively (Becton, Dickinson and Company, Franklin Lanes, NY, USA). These measurements were performed within 4 hours after blood drawing.

### Clinical and laboratory measurements

All samples were analyzed for serum apoA-I (Horiba ABX, Montpellier, France), apolipoprotein B-100 (apoB-100; Horiba ABX, Montpellier, France), high-sensitive C-reactive protein (hsCRP; Horiba ABX, Montpellier, France), total cholesterol (CHOD-PAP method; Roche Diagnostics, Mannheim, Germany), HDL cholesterol (precipitation method; Roche Diagnostics, Mannheim, Germany) and triacylglycerol with correction for free glycerol (GPO Trinder; Sigma-Aldrich Corp., St. Louis, MO, USA). LDL cholesterol was calculated by using the Friedewald formula [21]. Furthermore, fasting EDTA-plasma samples were analyzed for insulin concentrations (RIA; Millipore, Billerica, MA, USA) and fasting Na-F plasma samples were analyzed for glucose concentrations (Horiba ABX, Montpellier, France). IL-6, TNFα, E-selectin, thrombomodulin, P-selectin, ICAM-3, sICAM-1 and sVCAM-1 were measured by commercially available Multi Spot ELISA kits (Meso Scale Discovery, Rockville, MD, USA).

Liver and kidney function, and general health parameters (ureum, creatinin, alkaline phosphatase (ALP), γ-glutamyl transpeptidase (GGT), aspartate aminotransferase (ASAT), alanine aminotransferase (ALAT), total bilirubin, total protein, albumin, sodium, potassium, calcium, phosphorus and chloride) were measured in serum on a Beckman Coulter Synchron LX20 PRO Clinical System (Beckman Coulter Inc., Fullerton, CA, USA). Hematologic parameters (erythrocyte-, thrombocyte-, and leucocyte count, haemoglobin, haematocrit, erythrocyte mean corpuscular volume, erythrocyte mean corpuscular haemoglobin (concentration), and erythrocyte relative distribution weight) were measured on a Beckman Coulter LH750 (Beckman Coulter Inc., Fullerton, CA, USA) until October 2011. From October 2011 onwards, measurements were performed on a Sysmex XE-5000 System (Sysmex Corporation, Kobe, Japan). Haemostatic measurements (prothrombin time (PT) and activated partial thromboplastin time (aPTT)) were performed on a Sysmex CA-7000 Analyzer (Sysmex Corporation, Kobe, Japan).

Body weight was measured at each visit in the morning, after an overnight fast. Seated blood pressure was measured each visit (Omron M7, Omron Healthcare Co., Ltd, Kyoto, Japan), after 5 minutes rest. Measurements were performed four times and the average of the last three measurements is reported.

The degree of insulin resistance was estimated by HOMA_IR_, as described [22].

Compliance was checked by capsule count and by measuring total resveratrol and dihydroresveratrol concentrations (both free and conjugated forms) in plasma by liquid chromatography-mass spectrometry at day 28 of both intervention periods by DSM Nutritional Products, Ltd. Kaiseraugst, Switzerland.

### Statistical analyses

Before the start of the study, it was calculated that the statistical power to detect a true difference of at least 0.05 g/L in serum apo A-I concentrations between the experimental and control period was over 80%, when 45 subjects were included at P = 0.05. For the calculations, a within-subject variability of 0.12 g/L in serum apo A-I concentrations was used. As the expected drop-out rate was 10%, a total of 50 men and women were included.

A Shapiro-Wilk normality test was performed to examine if the changes between days 28 of the resveratrol and control periods were normally distributed. A student’s paired t-test was used to compare the values of the experimental and control periods from day 28 in normally distributed data. When data were not normally distributed, a Wilcoxon signed-rank test was used. Results, presented as means ± standard deviations (SD), were considered to be statistically significant if p < 0.05. Statistical analyses were performed using SPSS 19.0 for Mac Os X (SPSS Inc., Chicago, IL, USA).

## Results

### Subject characteristics and compliance

**Fig 1:**
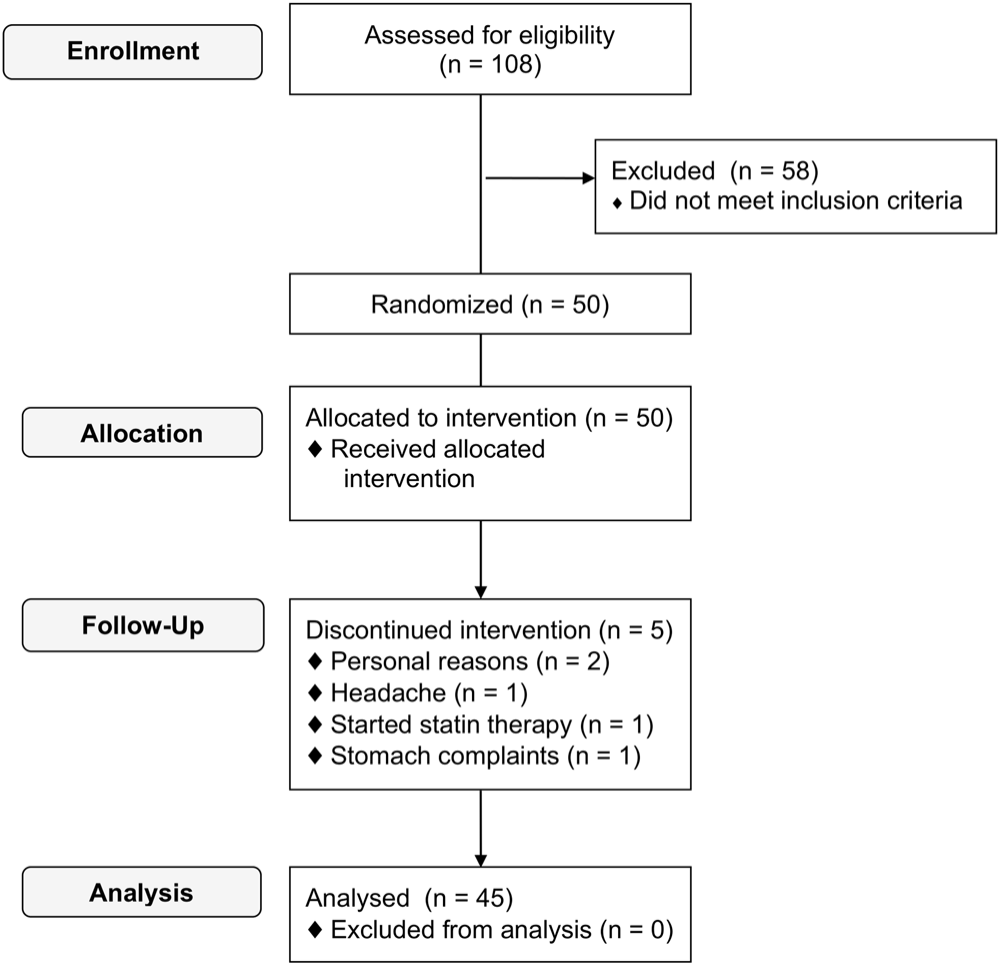
Subject flow chart.

A flow diagram of participants throughout the study is shown in Fig. 1. After screening, 50 subjects were eligible for participation and started the study. Two female subjects discontinued intervention because of personal reasons, 1 male subject dropped out because of a headache, one female subject dropped out because of stomach complaints and another female subject started statin treatment after randomization. A total of five subjects dropped out either during or straight after the first intervention period. Three of these subjects dropped out during the control period and two subjects during the resveratrol period. A total of 45 subjects (25 men and 20 women) completed the study. Baseline characteristics of study participants are shown in Table 1. Fourteen subjects used medication such as antidepressants, antihypertensives or antacids throughout the study without a change in dosage.

**Table 1:** Baseline characteristics of participants.^1,2^

	All (N = 45)	Males (N = 25)	Females (N = 20)
Age (y)	60 ± 7	62 ± 7	59 ± 7
BMI (kg/m^2^)	28.8 ± 3.2	28.5 ± 2.9	29.2 ± 3.6
Systolic BP (mmHg)	136 ± 17	138 ± 16	135 ± 18
Diastolic BP (mmHg)	88 ± 9	88 ± 10	88 ± 9
Heart rate (BPM)	67 ± 8	67 ± 8	68 ± 7
Glucose (mmol/L)	5.66 ± 0.53	5.71 ± 0.57	5.59 ± 0.48
Total cholesterol (mmol/L)	6.38 ± 1.00	6.20 ± 1.01	6.60 ± 0.97
HDL cholesterol (mmol/L)	1.14 ± 0.23	1.00 ± 0.17	1.30 ± 0.18

^1^Values are means ± SD.

^2^BP, blood pressure; BPM: beats per minute.

**Fig 2:**
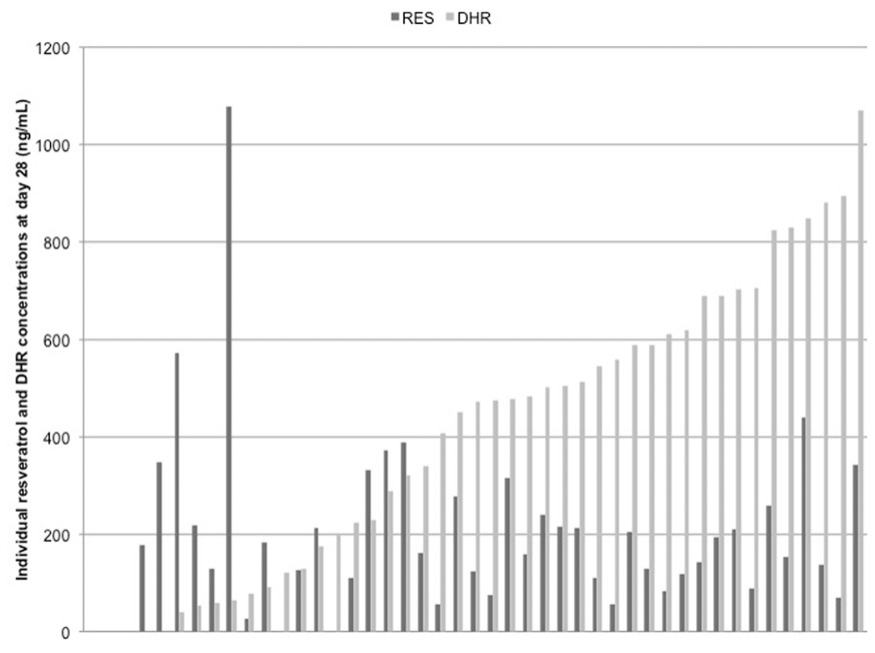
Individual total resveratrol (RES) and total dihydroresveratrol (DHR) concentrations at day 28.

As evidenced from pill count, compliance ranged between 88% and 111% and was on average 99% during both the control and resveratrol period. To further check for compliance, free and total (sum of conjugated and free resveratrol) resveratrol and dihydroresveratrol (DHR) concentrations were measured in all plasma samples. Free resveratrol and free DHR were not detectable, except in all plasma samples from one subject. Total resveratrol and DHR were detectable in three subjects during the control period and in one subject at the start of the resveratrol period. At day 28 of the resveratrol period, mean total resveratrol concentrations were 197 ± 185 ng/mL and those of DHR 408 ± 298 ng/mL. During the entire experimental period, one subject had no measurable plasma concentrations of total resveratrol or total DHR. Two subjects had no measurable resveratrol and DHR concentrations at day 28, while these compounds were present at day 25. Another two subjects had no measurable DHR concentrations during the total study period. For all subjects, plasma total resveratrol concentrations ranged between 0 and 1080 ng/mL, while total DHR concentrations varied between 0 and 1070 ng/mL during the resveratrol period. Individual resveratrol and DHR concentrations are shown in Fig. 2.

Composition of the diets between the periods was comparable (S1 Table). Body weight at the end of the control period was 84 ± 12 kg and at the end of the resveratrol period 84 ± 12 kg. These values were not significantly different (p = 0.910).

### Metabolic risk markers

The effects of 4-wk resveratrol supplementation on serum lipid and lipoprotein concentrations are presented in Table 2. No statistically significant differences were observed between the resveratrol and placebo periods on serum apoA-I and apoB-100 concentrations. In addition, no effects were found on serum total-, LDL- and HDL cholesterol and ratio of total to HDL-cholesterol. Triacylglycerol concentrations were significantly higher (P < 0.05) after the resveratrol period compared to the control period.

**Table 2:** Effect of 4-wk resveratrol intake on serum lipids and lipoproteins.^1^

	Placebo	Resveratrol	Difference	*P* value
Total cholesterol (mmol/L)	5.70 ± 0.90	5.69 ± 0.83	-0.02 ± 0.62	0.870
LDL cholesterol (mmol/L)	3.83 ± 0.84	3.77 ± 0.75	-0.06 ± 0.59	0.530
HDL cholesterol (mmol/L)	1.15 ± 0.23	1.15 ± 0.24	-0.01 ± 0.09	0.696
Total:HDL cholesterol ratio	5.14 ± 1.31	5.16 ± 1.24	0.02 ± 0.58	0.863
Triacylglycerol (mmol/L)^2^	1.58 ± 0.72	1.68 ± 0.77	0.10 ± 0.54	0.048
Apolipoprotein A-I (g/L)^2^	1.28 ± 0.19	1.27 ± 0.20	0.00 ± 0.12	0.670
Apolipoprotein B-100 (g/L)	1.17 ± 0.20	1.16 ± 0.17	-0.01 ± 0.11	0.545

Values are means ± SD.

^1^N = 45.

^2^These parameters were tested by a Wilcoxon signed-rank test for non-normal distributed data.

A significant increase in diastolic blood pressure and heart rate (P < 0.05 for both variables) was observed after the resveratrol period. Insulin concentrations, HOMA_IR_, systolic blood pressure and MAP were comparable between both periods (Table 3).

**Table 3:** Effect of 4-wk resveratrol intake on metabolic risk markers.^3^

BMI (kg/m^2^)^1^	28.3±3.1	28.4±3.1	0.0±0.5	0.760
Glucose (mmol/L) ^1,4^	5.30±0.54	5.22±0.49	-0.08±0.28	0.112
Insulin (mU/L) ^1^	13.1±5.0	12.9±5.3	-0.2±2.5	0.385
HOMA_IR_^1, 4^	3.13±1.35	3.03±1.44	-0.10±0.69	0.208
Systolic BP (mmHg) ^2, 4^	130±18	132±17	2±15	0.234
Diastolic BP (mmHg) ^2^	84±9	86±9	2±7	0.044
Heart rate (BPM) ^2^	64±8	67±8	3±7	0.025
MAP (mmHg) ^2, 4^	99±11	102±11	2±9	0.064

Values are means ± SD.

^1^N = 45.

^2^N = 44.

^3^BP, blood pressure; BPM, beats per minute; MAP, mean arterial pressure.

^4^These parameters were tested by a Wilcoxon signed-rank test for non-normal distributed data.

Table 4 shows the effect of resveratrol supplementation on markers for inflammation and endothelial function. No significant differences were found for hsCRP concentrations between both interventions. Also, no differences between both periods were found for IL-6, TNFα, E-selectin, thrombomodulin, P-selectin, ICAM-3, sICAM-1, and sVCAM-1 concentrations. Ten subjects (6 men and 4 women) had hsCRP levels > 10 mg/L on one or more occasions, which were equally divided across intervention and time points of blood sampling. Conclusions did not change when these subjects were excluded from analyses.

**Table 4:** Effect of 4-wk resveratrol intake on markers for inflammation and endothelial function.^1,2^

	Placebo	Resveratrol	Difference	*P* value
hsCRP (mg/L)^3^	2.64 ± 2.93	4.51 ± 10.24	1.87 ± 10.02	0.326
IL-6 (pg/mL) ^3^	1.63 ± 1.06	1.85 ± 1.42	0.23 ± 1.26	0.718
TNFα (pg/mL) ^3^	4.00 ± 1.26	3.95 ± 0.74	-0.05 ± 0.91	0.455
E-Selectin (ng/mL) ^3^	9.86 ± 6.51	9.64 ± 6.72	-0.22 ± 2.46	0.569
Thrombomodulin (ng/mL) ^3^	3.32 ± 0.77	3.29 ± 0.77	-0.03 ± 0.34	0.815
P-Selectin (ng/mL) ^3^	75 ± 68	73 ± 52	-2 ± 82	0.467
ICAM-3 (ng/mL) ^3^	0.67 ± 0.29	0.67 ± 0.30	0.00 ± 0.15	0.480
sICAM-1 (ng/mL)	324 ± 86	322 ± 82	-2 ± 64	0.829
sVCAM-1 (ng/mL)	561 ± 111	570 ± 130	8 ± 105	0.595

Values are means ± SD.

^1^N = 45.

^2^hsCRP, high-sensitivity C-reactive protein; ICAM-3, intercellular adhesion molecule-3; IL-6, interleukin-6; sICAM-1 soluble intercellular adhesion molecule-1; sVCAM-1, soluble vascular adhesion molecule-1.

^3^These parameters were tested by a Wilcoxon signed-rank test for non-normal distributed data.

Exploratory data analyses were performed to investigate the possible impact of subjects’ characteristics on the results. However, there were no indications that effects were different for men and women, or for obese (BMI ≥30 kg/m^2^; n = 16: 7 men and 9 women) and non-obese subjects (BMI <30 kg/m^2^; n = 29: 18 men and 11 women) separately. The same conclusion was drawn, when subgroups were formed based on median levels of day 0 of the control period for the HOMA_IR_ index, serum triacylglycerol concentrations, or on DHR concentrations or DHR:resveratrol ratios of day 28 of the resveratrol period. When analyses were restricted to subjects that did not use any prescribed medication (n = 31), the decrease in plasma glucose concentrations during the resveratrol period became statistically significant (-0.14 mmol/L; P < 0.05)

### General health parameters

Apart from statistically significant higher ALP values after resveratrol supplementation, no differences were found after 4-wk resveratrol treatment compared to control in any of the clinical chemistry, hematology and coagulation parameters (S2 Table).

## Discussion

In this study with overweight and slightly obese men and women, we found no effects on serum apoA-I and other metabolic risk markers after 4-wk of daily supplementation with 150 mg *trans*-resveratrol. Compliance of the subjects was excellent, as indicated by capsule counts and total plasma resveratrol concentrations. A lack of effect on apoA-I concentrations does not support *in vitro* data, which suggests that resveratrol induces the expression of the apoA-I gene [14,23]. So far, however, no human studies have specifically addressed the effects of *trans*-resveratrol on apoA-I concentrations. Our finding that resveratrol supplementation also has no effect on HDL cholesterol agrees with the results of a very recent meta-analysis. In that study, no effects of resveratrol on HDL cholesterol concentrations were found, irrespective of dose, study duration, and cardiovascular risk status of the subjects [24]. A major limitation of this meta-analysis was that, in four out of the seven studies included, subjects used cholesterol-lowering and/or hypoglycemic drugs, while in none of the studies changes in serum lipid concentrations were the primary outcome parameter. In our study, however, subjects did not use these types of medication and were selected based on low HDL cholesterol concentrations. In addition, in only five of the seven studies, purified *trans*-resveratrol was used, whereas in the other studies resveratrol-enriched grape extract or *Polygonum Cuspidatum* extract containing *trans-*resveratrol was given. However, like in the recent meta-analysis, we also did not observe any effects on serum total cholesterol, LDL cholesterol and in addition apoB100 concentrations. Another recent double-blind cross over trial tested the effects of high-dose (e.g. 1000 mg during the first week and 2000 mg during the second week) *trans-*resveratrol intake on apoB100 production- and catabolic rate in overweight and obese, mildly hypertriglyceridemic males. Reductions of both apoB100 production and catabolic rates were found without accompanying changes in plasma triglyceride-rich lipoprotein (TRL) apoB100 concentrations [25]. This may agree with our results, although it should be noted that the major of the plasma apoB100 pool is transported by the triglyceride-poor LDL particles. To summarize, it is not very likely that the putative positive effects of resveratrol on cardiovascular health are mediated by changes in serum lipid or lipoprotein concentrations.

The question then remains whether resveratrol protects against cardiovascular disease through effects other than those on lipid and lipoprotein metabolism. Several studies have reported positive effects of resveratrol on insulin sensitivity, accompanied by a significant lowering of blood glucose levels. However, we did not observe a statistically significant change in HOMA_IR_ and a tendency towards decreased fasting plasma glucose concentrations, which became statistically significant, when subjects who used medication were excluded from the statistical analyses. Results of resveratrol on plasma glucose concentrations are conflicting, and both positive and no-effect studies have been reported. Again, studies have been performed in diverse populations. Effects on fasting glucose concentrations have mainly been found in studies with type 2 diabetes mellitus (T2DM) patients [26,27], although part of these results have to be interpreted cautiously because of methodological constraints [27]. No effects have been found in a study that included obese, but otherwise healthy subjects [18]. Two other studies used the same type of *trans*-resveratrol as we did [19,20]. In one study, no effect on fasting glucose concentrations was found after 12 weeks of 75 mg/d resveratrol supplementation in non-obese women with normal glucose tolerance [20], whereas Timmers et al. reported a significant decrease of 0.18 mmol/L in glucose after 30-day resveratrol supplementation in obese, but healthy male subjects [19]. Although changes in glucose concentrations were a secondary outcome, our study had a statistical power of nearly 100% to detect a true change in glucose of 0.18 mmol/L at an alpha of 0.05. Thus, daily supplementation with 150 mg *trans*-resveratrol may improve glucose metabolism, but more well-designed studies are needed to answer this question.

*In vitro* and animal studies have suggested positive effects of resveratrol on endothelial function and low-grade systemic inflammation [28]. However, our results are in line with other human studies that did not show an effect on different markers for low-grade inflammation, such as CRP, IL1β, IL6, IL8, TNFα and monocyte chemotactic protein-1 [18–20,26,29]. In one study, a reduction in TNFα [19] was found, which we—and others [18]—could not reproduce. Regarding vascular and endothelial function, Agarwal et al. showed a lower expression of VCAM- and ICAM mRNA after treatment of human coronary artery endothelial cells with plasma from subjects treated with resveratrol [29]. However, we found no effects on circulating levels of ICAM-3, sICAM-1, sVCAM-1, E-Selectin and P-Selectin. Therefore, consistent evidence from human studies for beneficial effects of resveratrol intake on plasma markers for low-grade inflammation and endothelial function is missing.

Finally, it has been suggested that *trans*-resveratrol lowers systolic blood pressure (SBP) and mean arterial pressure (MAP) [19]. However, these effects could not be confirmed in other studies, which also found no effects on diastolic blood pressure (DBP) and heart rate [18,20,30]. In our study, SBP and MAP also remained unchanged, but DBP and heart rate increased after *trans*-resveratrol intake, for which we have no explanation.

It is possible that certain groups may benefit from resveratrol supplementation and other groups do not. Although our study was not powered to answer this question adequately, exploratory data analyses did not suggest that effects did depend on sex, BMI, triacylglycerol concentrations or HOMA_IR_. Interestingly, Bode et al. showed pronounced inter-individual differences in *trans-*resveratrol metabolism by human gut microbiota [31], which might also contribute to discrepancies between studies. Also, in our subjects we found large differences in plasma DHR concentrations. However, we found no indications that results did depend on DHR concentrations or DHR:resveratrol ratios.

In summary, we here show that 150 mg of daily resveratrol intake for four weeks does not change lipids, lipoproteins and other metabolic risk markers related to cardiovascular health in overweight and slightly obese men and women with low HDL cholesterol concentrations. Effects on glucose metabolism however warrant further study.

## Supporting Information

S1 CONSORT Checklist (DOC)

S1 Protocol (DOCX)

S1 Table Dietary intake during the placebo and resveratrol period, as estimated with food-frequency questionnaires. (DOCX)

S2 Table Effect of 4-wk resveratrol intake on hematologic, hemostatic and general health parameters. (DOCX)
