# Supplementary material for: Resveratrol Does Not Influence Metabolic Risk Markers Related to Cardiovascular Health in Overweight and Slightly Obese Subjects: A Randomized, Placebo-Controlled Crossover Trial
Source: PLoS One. 2015 Mar 19;10(3):e0118393. doi: 10.1371/journal.pone.0118393 (PMC4366169; doi:10.1371/journal.pone.0118393)
Supplement: S1 Protocol — (DOCX) [file pone.0118393.s002.docx]

De effecten van resveratrol op serum apolipoproteïne A-I concentraties in mannen en vrouwen met verlaagde HDL-cholesterol concentraties

**The effects of resveratrol on serum apolipoprotein A-I concentrations in men and women with low HDL-cholesterol concentrations**

**Written by:**

Sanne van der Made, MSc

Jogchum Plat, PhD

Ronald P. Mensink, PhD, Msc

Department of Human Biology

School for Nutrition, Toxicology and Metabolism

Maastricht University

P.O. Box 616

6200 MD Maastricht

The Netherlands

Telephone: +31-43-3881742

FAX: +31-43-3670976

E-mail: R.Mensink@HB.UNIMAAS.NL

**PROTOCOL TITLE**

De effecten van resveratrol op serum apolipoproteïne A-I concentraties in mannen en vrouwen met verlaagde HDL-cholesterol concentraties

**The effects of resveratrol on serum apolipoprotein A-I concentrations in men and women with low HDL-cholesterol concentrations**

| **Protocol ID** | prmec022 |
| --- | --- |
| **Short title** | Resveratrol and serum apo A-I |
| **Version** | 5.0 |
| **Date** | March 20, 2012 |
| **Coordinating investigators/project leaders** | Sanne van der Made, MSc  Jogchum Plat, PhD  Ronald P. Mensink, PhD  Department of Human Biology  NUTRIM School for Nutrition, Toxicology and Metabolism  Maastricht University  P.O. Box 616  6200 MD Maastricht  The Netherlands  Telephone: +31-43-3881742  Fax: +31-43-3670976  E-mail: R.Mensink@HB.unimaas.nl |
| **Independent physician(s)** | B. Groen, MD  Department of Movement Sciences  Universiteitssingel 50  6229ER Maastricht  The Netherlands  Tel. 043-3881390  E-mail: bart.groen@maastrichtuniversity.nl |

**PROTOCOL SIGNATURE SHEET**

| **Name** | **Signature** | **Date** |
| --- | --- | --- |
| **Principal investigators:**  **J. Plat, PhD**  **R.P. Mensink, PhD** |  |  |

**TABLE OF CONTENTS**

LIST OF ABBREVIATIONS AND RELEVANT DEFINITIONS 6

SUMMARY 7

1. INTRODUCTION AND RATIONALE 8

2. OBJECTIVES 9

3. STUDY DESIGN 9

3. STUDY POPULATION 11

3.1 Population 11

3.2 Inclusion criteria 12

3.3 Exclusion criteria 12

3.4 Sample size calculation 13

4. TREATMENT OF SUBJECTS 13

4.1. Investigational Product and supplementation regimen 13

4.1.1 Investigational Product 13

4.1.2 Randomization, blinding and treatment allocation 14

4.1.3 Supplementation regimen 14

4.1.4 Dispensing and accountability 15

4.1.5 Compliance 15

4.1.6 Concomitant treatments/supplements and restrictions 15

4.2 Postprandial test 16

4.2.1 Composition of the postprandial menu 16

4.2.2 Preparation and composition of the meals 17

5. METHODS 17

5.1 Study procedures 17

5.2 Study parameters/endpoints 18

5.2.1 Metabolic risk markers 18

5.2.3 Pulse wave velocity 19

5.2.4 Retinal images 19

5.2.5 Clinical Chemistry 19

5.2.6 Additional parameters 21

5.2.6.2 Body weight 21

5.2.6.3 Resveratrol concentration 21

5.3 Withdrawal of individual subjects 22

5.4 Replacement of individual subjects after withdrawal 23

5.5 Follow-up of subjects withdrawn from treatment 23

6. SAFETY REPORTING 23

6.1 Section 10 WMO event 23

6.2 Adverse and serious adverse events 23

6.2.1 Suspected unexpected serious adverse reactions (SUSAR) 24

6.2.2 Adverse event assessment 24

6.2.3 Adverse event reporting 25

6.2.4 Follow-up of adverse events 25

7. STATISTICAL ANALYSIS 25

7.1 Descriptive statistics 25

7.2 Univariate analysis 26

8. ETHICAL CONSIDERATIONS 26

8.1 Regulation statement 26

8.2 Recruitment and consent 26

8.3 Privacy 26

8.4 Benefits and risks assessment, group relatedness 26

8.5 Compensation for injury 27

8.6 Incentives 27

9. ADMINISTRATIVE ASPECTS AND PUBLICATION 28

9.1 Handling and storage of data and documents 28

9.2 Amendments 28

9.3 End of study report 28

9.4 Public disclosure and publication policy 28

10. REFERENCES 29

Appendices:

1. Composition and safety statement capsules
2. Investigator Brochure
3. SAE and Pregnancy forms
4. SAE form completion guideline
5. SAE reporting flow-chart

# LIST OF ABBREVIATIONS AND RELEVANT DEFINITIONS

| **ApoA-I** | Apolipoprotein A-I |
| --- | --- |
| **ApoB48** | Apolipoprotein B48 |
| **ABCA1** | ATP binding cassette transporter 1 |
| **CCMO** | Central Committee on Research Involving Human Subjects |
| **CHD** | Coronary Heart Disease |
| **CV** | Curriculum Vitae |
| **FMD** | Flow mediated vasodilatation |
| **HDL-C** | High-density lipoprotein cholesterol |
| **hsCRP** | High-sensitive C-reactive protein |
| **IL-6** | Interleukin-6 |
| **LDL-C** | Low-density lipoprotein cholesterol |
| **MCP-1** | Monocyte chemotactic protein-1 |
| **METC** | Medical research ethics committee (MREC); in Dutch: medisch ethische toetsing commissie (METC) |
| **PPAR** | Peroxisome proliferator-activated receptor |
| **PWV** | Pulse wave velocity |
| **(S)AE** | (Serious) Adverse Event |
| **sE-Selectin** | Soluble E-Selectin |
| **sICAM-1** | Soluble Intercellular adhesion molecule 1 |
| **VCAM-1** | Vascular cell adhesion molecule 1 |
| **Sponsor** | The sponsor is the party that commissions the organisation or performance of the research, for example a pharmaceutical company, academic hospital, scientific organisation or investigator. A party that provides funding for a study but does not commission it is not regarded as the sponsor, but referred to as a subsidizing party. |
| **TAG** | Triacylglycerol |
| **TCH** | Total cholesterol |
| **WMO** | Medical Research Involving Human Subjects Act (Wet Medisch-wetenschappelijk Onderzoek met Mensen |

#

# SUMMARY

**Rationale**: A substantial residual cardiovascular risk is still present, despite successful efforts to lower atherogenic serum low-density lipoprotein cholesterol (LDL-C) concentrations. An additive strategy may be raising high-density lipoprotein (HDL) concentrations, and in particular those of its major protein constituent apolipoprotein A-I (apoA-I). Based on cell and animal studies, resveratrol may be a promising candidate in this respect. Effects of this dietary component on apoA-I has however never been evaluated in a placebo controlled human intervention study.

**Objective**: To evaluate the effects of resveratrol on apoA-I concentrations in subjects with low serum HDL-cholesterol (HDL-C) concentrations at baseline. Minor objectives are to study the effects of resveratrol on (1) endothelial function and arterial stiffness as measured by flow mediated vasodilatation (FMD) and pulse wave velocity (PWV) during the fasting and postprandial phase (2) endothelial function of the retinal vasculature (3) lipid and glucose metabolism during the fasting and postprandial phase, and (4) biomarkers for low-grade systemic inflammation (interleukin-6 (IL-6), C-reactive protein (CRP)) and endothelial function (monocyte chemotactic protein-1 (MCP-1), sE-selectin, soluble intercellular adhesion molecule 1 (sICAM-1), and soluble vascular cell adhesion molecule-1 (sVCAM-1)).

**Study design**: A randomized, double-blind, placebo controlled cross-over design. The total study duration will be 12 weeks, consisting of a 4 weeks experimental period, a 4 weeks wash-out and a 4 weeks control period.

**Study population**: Fifty overweight and obese men and women, aged 45-70 years, with low HDL-C concentrations (men <1.0 mmol/L and women <1.3 mmol/L).

**Intervention**: During the experimental period, subjects will receive daily one capsule at lunch and one capsule at dinner, each providing 75 mg resveratrol. During the control period, subjects will receive daily two placebo capsules, each containing 58 mg cellulose.

**Main study parameters/endpoints**: Measurements will be performed at the start and end of each 4-week intervention period. Effects of resveratrol supplementation will be calculated as the absolute differences between values obtained at the end of each period. The primary endpoint is the change in serum apoA-I concentrations.

**Nature and extent of the burden and risks associated with participation, benefit and group relatedness**: Before the study, subjects will be screened to determine eligibility during two visits of respectively 15 and 10 minutes. During these visits, body weight, height and blood pressure will be measured. In addition, a venous blood sample (5.5 mL at each occasion) will be drawn. During the study, subjects will receive the control and resveratrol capsules in random order. At days 1, 25, and 28 of each 4 wk experimental period, a fasting blood sample will be drawn (2 x 10 mL and 4 x 16.5 mL). In addition, a 4 hr postprandial test will be performed at day 28 of each experimental period. For this, subjects will receive two muffins and 300 mL low-fat milk, and an additional 8 blood samples will be taken (80 mL at each test). Thus, in total 257 mL blood will be drawn. Before and after each postprandial test, two FMD (including an ECG) and two PWV measurements will be performed. During the last week of each intervention period, a retinal image will be taken on a separate occasion. Subjects will be asked to fill out a food frequency questionnaire two times and to keep a study-diary throughout 12 weeks. On rare occasions, blood sampling might cause bruises or hematoma. Total time investment for the subjects will be approximately 16 hours.

# 1. INTRODUCTION AND RATIONALE

Despite aggressive treatment regimens to lower atherogenic low-density lipoprotein cholesterol (LDL-C) concentrations below 2.6 mmol/L, there is still a substantial residual cardiovascular risk. A wealth of evidence from in vitro and in vivo studies, however, shows that raising high-density lipoprotein cholesterol (HDL-C) or apolipoprotein A-I (apoA-I) concentrations protects against coronary heart disease (CHD). For example, increasing serum apoA-I and/or HDL-C concentrations by intravenous infusion of recombinant apoA-I Milano / phospholipid complexes rapidly reduced atheroma-volume in patients with acute coronary syndromes (1). Also, selective delipidation of plasma HDL (converting large HDL-particles to small pre-beta HDL-particles, thereby increasing the relative amount of apoA-I) enhanced reverse cholesterol transport in vivo in African Green monkeys (2). This suggests that increasing apoA-I concentrations may be even more beneficial than simply increasing the HDL-C pool. In addition, apoA-I protein may have anti-inflammatory, antioxidant, and anti-thrombotic effects, which may even enhance its cardioprotective effects (3-5). In fact, a special working group already emphasized in 2001 the need to develop interventions to raise HDL-C or apoA-I (6). However, despite many initiatives, we have to admit that 9 years later no new widely applicable intervention strategies with proven efficacy have been developed. This is even more worrisome since the number of subjects with obesity, type II diabetes and the metabolic syndrome - for which dyslipidemia (low HDL-C and high triacylglycerol) is an important clinical hallmark - has increased steadily. Therefore, there is strong need to better understand possibilities to intervene with apoA-I and HDL synthesis.

Epidemiological studies have now shown that a higher polyphenol intake is associated with a reduced cardiovascular risk (7, 8). This association may be due to increased intakes of resveratrol, a polyphenol found in food items such as red wine and grapes. It has been found that resveratrol influences numerous mechanisms, which act on the progression as well as on the regression of atherosclerosis (9). In fact, in hyperlipidemic genetically modified animal models, resveratrol-enriched diets suppressed lesion formation (10, 11). In addition, human intervention studies showed a HDL-C elevating effect of berries (12) and red wine (13), both of which contain resveratrol. The amount of resveratrol in the diet is low. Concentrations in red wine, for example, vary from undetectable to 14.3 mg/L (14). Resveratrol however is safe and no serious adverse events were noted after single oral doses of 1, 2.5 or 5 g resveratrol in humans (15).

The production of apoA-I, the predominant apoprotein in HDL particles is under direct PPAR alpha transcriptional control. The positive effects of fibrates - known PPARalpha ligands - on serum apoA-I and HDL-C concentrations are also ascribed to their PPAR alpha activating potential (16). In this respect, it is an important finding that resveratrol can elevate the activity of the transcription factor PPAR alpha, a finding that has been patented (17, 18). In this patent, it is described that in vitro resveratrol increases apoA-I production via PPAR alpha activation. By using the newly developed drug RVX-208, a resveratrol derivate, which is based on this patent and targets de novo apoA-I production, a significant increase in serum apoA-I concentrations, predominantly in the pre-beta HDL fraction was shown in African Green monkeys (19). Importantly, also the cholesterol efflux from cholesterol loaded macrophages towards the HDL fraction via the ABCA1 pathway was improved. As far as we know, these effects have not been studied in humans. We therefore propose to evaluate, in a placebo controlled double-blind human intervention study, the effects of resveratrol in subjects with low serum HDL-C concentrations on changes in serum apoA-I and HDL-C (subfraction) concentrations. Moreover, we will also examine functional effects due to resveratrol supplementation by measuring the capacity of the vessels to respond to a standardized fat-load during the postprandial period by measuring flow-mediated vasodilatation (FMD) of the brachial artery and arterial stiffness by pulse wave velocity (PWV). Noteworthy, with the first technique it has earlier been shown that infusion of HDL improved endothelial function (20).

# 2. OBJECTIVES

Considering the background information given in the previous paragraph, the following research questions are formulated:

**Primary research question:**

What is the effect of resveratrol supplementation on serum apoA-I concentrations in men and women with low HDL-C concentrations?

*Major null hypothesis, H_0_:*

As compared with control capsules, a daily intake of two capsules each containing 75 mg resveratrol for 4 weeks does not change serum apoA-I concentrations in subjects with low HDL-C concentrations.

*Major alternate hypothesis, H_a_:*

As compared with control capsules, a daily intake of two capsules each containing 75 mg resveratrol for 4 weeks does change serum apoA-I concentrations in subjects with low HDL-C concentrations.

**Secondary research questions:**

1. What is the effect of resveratrol supplementation on FMD and PWV during the fasting and postprandial phase?
2. What is the effect of resveratrol supplementation on retinal microvasculature, measured as AV-ratio and vessel diameter from digitized analogue fundus photographs?
3. What is the effect of resveratrol supplementation on lipid (apoB, total cholesterol, LDL-cholesterol, HDL-C and triglycerides) and glucose (glucose and insulin) metabolism during the fasting and postprandial phase?
4. What is the effect of resveratrol supplementation on serum biomarkers for low-grade systemic inflammation (hsCRP, IL-6) and endothelial function (sICAM-1, VCAM-1, MCP-1 and sE-Selectin)?

# 3. STUDY DESIGN

A randomized, double-blind, placebo-controlled cross-over study will be carried out, as outlined in Table 1.

**Duration of the study**

It is projected that the study will start October 1, 2010. It is expected that the last subject will finish the study June 30, 2011.

Each subject will be involved in the study for 14 weeks:

- 1-2 weeks screening before enrolment
- First period: 4 weeks
- Wash-out period: 4 weeks (21, 22)
- Second period: 4 weeks

Table 1: Experimental design

|  | Screening | | Intervention period | | | Wash-out period | Intervention period | | |
| --- | --- | --- | --- | --- | --- | --- | --- | --- | --- |
| Group 1 (N=25) |  | | Experimental capsules | | |  | Placebo capsules | | |
| Group 2 (N=25) |  |  | Placebo capsules | | |  | Experimental capsules | | |
| Week |  | | 1 | 4 | | 4 weeks | 9 | 12 | |
| Day |  |  | 0 | 25 | 28 |  | 56 | 81 | 84 |
| Visit | -2 | -1 | 1 | 2 | 3 |  | 4 | 5 | 6 |
| Blood sampling | X | X | X | X | X |  | X | X | X |
| Postprandial test |  |  |  |  | X |  |  |  | X |
| Flow mediated dilation, including ECG |  |  |  |  | XX |  |  |  | XX |
| Pulse wave velocity |  |  |  |  | XX |  |  |  | XX |
| Retinal images * |  |  |  | X | |  |  | X | |
| Body weight | X | X | X | X | X |  | X | X | X |
| Height | X |  |  |  |  |  |  |  |  |
| Blood pressure,  heart rate | X | X | X | X | X |  | X | X | X |
| Capsule count |  |  |  | X | X |  |  | X | X |
| Food intake |  |  |  |  | X |  |  |  | X |
| Study diary |  |  | During total study period |  |  |  |  |  |  |
| AE reporting |  |  | During total study period | | | | | | |

* At an extra visit during the last week of each experimental period

Before screening, subjects will be informed about the procedures and informed consent will be obtained. Subjects will be informed about their results and advised to consult their general practitioner when values are outside normal ranges. When a subject does not want to be informed about the results, it is not possible to participate in this study. Subjects that fulfil all inclusion criteria and are willing to participate will be randomly allocated to receive either the placebo capsules or the experimental capsules for a period of four weeks. This period will be followed by a four-week wash-out period, after which regimes will be crossed over. In this way variation due to drift of variables with time is eliminated. Possible residual effects will be minimized by a washout period of at least four weeks between two periods. The placebo and experimental capsules have to be consumed at lunch and dinner, and will be coded to assure blindness of the investigator and participants. Experimental capsules will each contain 75 mg resveratrol and placebo capsules 58 mg cellulose (**Appendix 1**). During each visit, subjects will be supplied with the amount of capsules that is sufficient for the next visit. Leftovers should be returned and counted.

During the study, subjects will record in diaries any signs of illness, medication used, menstrual phase and any deviations from the protocol. Subjects are asked not to change their dietary habits, except for stopping the use of resveratrol-containing products during the study. Subjects will receive a list in which the resveratrol-containing products are stated, examples are wine, grapes and berries. Furthermore, subjects will be asked to maintain their level of physical exercise and use of oral contraceptives. At the end of each intervention period, food intake will be assessed by a validated food frequency questionnaire. Fasting blood samples will be drawn before the start of the study (day 0), twice in week 4 (days 25 and 28), after 8 weeks (day 56) and twice at the end of the study (days 81 and 84). FMD and PWV measurements will be performed at the end of each four-week intervention period (days 28 and 84), before and 4 hours after a standardized fat-load. During these days, blood will be sampled as indicated in figure 1 and as further explained in paragraph 6.2. Furthermore, retinal images will be made during the last week of each experimental period (see paragraph 6.3.2 and 6.3.3). Body-weight, height, blood pressure and ECG will be recorded as indicated in Table 1.

# 3. STUDY POPULATION

## 3.1 Population

Men and women, aged between 45 and 70 years, will be recruited. The lower limit for age is set at 45 years, because from experiences in the past it became clear that the major part of people aged under 45 will not meet the inclusion criteria as formulated in §3.2. The upper limit for age is set at 70 years, because from our previous experience a large proportion of people aged over 70 years will not meet the inclusion criteria as formulated in paragraph 4.2. Because we do not want to encumber these subjects unnecessarily, we have decided to set the lower limit for age at 45 years and the upper limit at 70 years.

## 3.2 Inclusion criteria

Subjects are asked to fill out a general health questionnaire in between the first and second screening visit. Only healthy subjects will be included. The inclusion criteria are:

- aged between 45 and 70 years
- HDL-C <1.21 mmol/L (men)
- HDL-C <1.53 mmol/L (women)
- serum total cholesterol <8.0 mmol/L
- plasma glucose <7.0 mmol/L
- BMI between 25 – 35 kg/m^2^
- non-smoking
- willingness to abstain from resveratrol rich products from two weeks prior to the study and the duration of the study:
  - grapes
  - wine (red and white)
  - all berries
  - peanuts
  - peanut butter
  - soy (products)
  - pomegranate
  - grape juice
  - port wine

## 3.3 Exclusion criteria

The exclusion criteria are:

- unstable body weight (weight gain or loss >3 kg in the past 3 months)
- any medical condition requiring treatment and/or medication use
- indication for treatment with cholesterol-lowering drugs according to the Dutch Cholesterol Consensus
- use of medication or a medically-prescribed diet known to affect serum lipid or glucose metabolism. The use of oral contraceptives and paracetamol is allowed.
- Active cardiovascular disease (for instance congestive heart failure) or recent (<6 months) event, such as acute myocardial infarction or cerebro-vascular accident
- not willing to stop the consumption of vitamin supplements, fish oil capsules or products rich in plant stanol or sterol esters 3 weeks before the start of the study
- men: consumption of >21 glasses of alcohol-containing drinks per week

women: consumption of >14 glasses of alcohol-containing drinks per week

- abuse of drugs
- pregnant or breastfeeding women
- participation in another biomedical study within 1 month prior to the screening visit
- having donated blood (as blood donor) within 1 month prior to the screening visit, planning to donate blood during the study or within one month after finishing the study
- impossible or difficult to puncture as evidenced during the screening visits

## 3.4 Sample size calculation

The statistical power to detect a difference of at least 50 mg/L in serum apo A-I concentrations between the experimental and control period is over 80%, when 45 subjects are included at P=0.05. For the calculations, a within-subject variability of 120 mg/L in serum apo A-I concentrations is used. As the expected drop-out rate is 10%, a total of fifty men and women will be recruited.

# 4. TREATMENT OF SUBJECTS

For the first four weeks of the study, subjects will be randomly allocated to either the experimental or the control group. Capsules should be taken twice a day, i.e. one capsule at lunch and one capsule at dinner. After a 4 weeks of wash-out period, during which no capsules should be taken, regimes will be crossed over. Experimental capsules will contain 75 mg resveratrol, placebo capsules will contain 58 mg cellulose. Thus, either 150 mg resveratrol or 116 mg cellulose will be consumed each day.

## 4.1. Investigational Product and supplementation regimen

### 4.1.1 Investigational Product

#### 4.1.1.1 Investigational product name and formulation

Resveratrol will be provided as trans-Resveratrol (resVida^TM^), provided as capsules (75 mg). DSM Nutritional Products Ltd (DNP) will provide the study investigational product (resVida™) and placebo capsules.

- Product name: resVida™

- Active compound: *trans*-Resveratrol

- Formulation: 99% pure, crystalline.

- Lot: RC0908009

- Capsules: 75 mg active compound/capsule

#### 4.1.1.2 Packaging and labelling

The product will be packaged and labelled by the principle investigators.

The label on the investigational product bears the following information:

- Investigator name, address, phone number
- DSM Nutritional Products Ltd.
- Dosage form, route of administration, quantity of dosage units
- Protocol number
- Investigational product Batch number
- Subject ID
- Storage conditions
- Keep out of reach of children
- For investigational use only
- Directions of use
- Expiration date

#### 4.1.1.3 Handling and storage conditions

The study capsules will be distributed into study bottles and dispensed to study subjects. The capsules will be stored in the dark at room temperature in room 3.247, Universiteitssingel 50, Maastricht.

### 4.1.2 Randomization, blinding and treatment allocation

The subjects will receive the two types of capsules in random order, based upon a computer-generated table with random numbers. For this, a categorical list in logical order will be created by an independent person. This list will include the two interventions for each subject. After addition of a computer-generated list of random numbers, the list will be sorted by subject number and random number, resulting in a randomized list of treatment allocation. To blind the researchers and the subjects, the capsules will be coded. The randomization code will be broken after statistical analyses are completed.

### 4.1.3 Supplementation regimen

#### 4.1.3.1 Rationale for dose selection

For the product resVida^TM^ the maximal recommended daily dosage in humans is 150 mg (investigator brochure DSM page 33). Therefore, we have chosen to supplement the subjects with a dose of 150 mg daily, spread out over doses of 75 mg twice a day with lunch and diner, this because the bioavailability of resveratrol is rather low (23). Detailed information on resVida^TM^ can be found in the “investigator brochure” **(Appendix 2)** enclosed with the protocol) supplied by DSM, which contains general information on resVida^TM^ (investigator brochure page 5-8), information on toxicology, pharmacokinetics, and metabolism of resVida^TM^ in animals (investigator brochure page 9-26), and the effects of resVida^TM^ supplementation in humans (metabolism, safety and pharmacokinetics) (investigator brochure page 28-33). The brochure is composed of mixture of research performed within DSM itself, and peer-reviewed scientific articles.

#### 4.1.3.2 Dosage regimen

Daily intake of two capsules at lunch and dinner each containing 75 mg resveratrol (=150 mg resveratrol per day) or placebo for 4 weeks.

First intake first period: on day 0 after the measurements and blood sampling

4-weeks supplementation until day 27

Last intake first period: day 27 evening

First intake second period: on day 56 after the measurements and blood sampling

4-weeks supplementation until day 83

Last intake second period: day 83 evening

No intake on the days when retinal images are taken (last week of each experimental period) and the postprandial tests (day 28 and day 84) are performed.

#### 4.1.3.3 Route of administration

The capsules will be taken orally.

#### 4.1.3.4 Supplementation duration

Four weeks of supplementation during the first experimental period and four weeks of supplementation during the second experimental period separated by a wash-out period of 4 weeks with no supplementation.

###

### 4.1.4 Dispensing and accountability

If applicable, the investigator will receive a Product Dispensing and Return Log in order to account for investigational product dispensed and returned by the subject. It must be kept up-to-date and list the subject ID, the amount of investigational product and date dispensed to the subject and the amount of product and date returned. Subjects must be instructed to return unused product on each visit.

The investigator must return all unused products to DNP or discard as agreed with the Study Director at the end of the study.

###

### 4.1.5 Compliance

At the end of each supplementation period, the study personal will perform a pill count. Subjects will be considered to be compliant with the study regimen if they take 80% of the planned supplementation.

###

### 4.1.6 Concomitant treatments/supplements and restrictions

If treatment for an adverse event becomes necessary, the medication(s) will be reported on the concomitant medication section of the case report form (CRF), including generic name, indication, total daily dose, route and time/duration of administration.

Allowed concomitant drug intake:

Short-term (max 1 week) during the study period: unspecific, peripheral analgesics, beta-lactam antibiotics

Subjects will be asked not to take any supplements beginning two weeks before and continuing throughout the entire study.

General lifestyle including dietary habits (i.e. excluding resveratrol containing food items) and physical activity should be maintained throughout the study.

## 4.2 Postprandial test

At the end of each intervention period (days 28 and 84), subjects will participate in a postprandial test. On the day preceding the test day, subjects will be asked to avoid high fat foods, alcohol, caffeine and taking part in any strenuous activity / exercise. On the morning of the test - after a 12 hr overnight fast (from 8 PM) - subjects are allowed to drink a glass of water in the morning. After arrival, subjects will be weighed and have to rest for 15 minutes in the supine position. Then, the first ECG, FMD and PWV measurements will be performed. After these first measurements, a venous cannula will be inserted and a fasting blood sample (t=0, 16.5 mL) will be collected. Subjects are then requested to consume, within 10 minutes, two muffins and 300 mL low-fat milk (see Tables 2 and 3). Subsequent blood samples are collected via the cannula at 15, 30, 45, 60, 90, 120 and 240 minutes (each 16.5 mL), as indicated in figure 1. After the last blood sample is taken, the cannula will be removed, a second FMD and PWV measurement is started. During the 4-hour period, subjects are allowed to drink water. This protocol will be followed during both postprandial test days (days 28 and 84).

### 4.2.1 Composition of the postprandial menu

The test meal will consist of two muffins and 300 mL low-fat milk, which will provide 4.6 MJ (4598 kJ or 1100 kcal): 56.6 g fat, 53.5 g protein and 121 g carbohydrate (macronutrient composition of the postprandial menu is given in Table 2).

Table 2: Macronutrient composition of 2 muffins and 300 mL low-fat milk

| Nutrient | Muffins | Low-fat milk | Total |
| --- | --- | --- | --- |
| Energy (kJ) | 4095 | 503 | 4598 |
| Energy (kcal) | 980 | 120 | 1100 |
| Protein (g) | 41.5 | 12 | 53.5 |
| Carbohydrates (g) | 103 | 18 | 121 |
| Total fat (g) | 56.6 |  |  |
| Saturated fatty acids (g) | 33.9 |  |  |
| Trans fatty acids (g) | 2.2 |  |  |
| Monounsaturated fatty acids (g) | 14.5 |  |  |
| Polyunsaturated fatty acids (g) | 2.7 |  |  |
| Linoleic acid | 1.7 |  |  |
| Cholesterol (mg) | 349 |  |  |

### 4.2.2 Preparation and composition of the meals

The ingredients needed to yield 2 muffins are: butter (61.7 g), flower (60 g), sugar (60 g) and 1 egg (60 g), as shown in table 3.

Table 3: Muffin ingredients, brands and place to buy ingredients and milk

Muffins will be baked at the Department of Human Biology by our research dietician using standard ingredients that will be bought at the local supermarket. For preparation of the muffins, the batter is transferred into muffin cases by weighing out a 90 g portion for each muffin. Before preparing the muffins, our dietician will check the shelf life of all ingredients. The muffins are baked at 180 °C, in a fan-assisted oven for 20 minutes. After cooling down, the muffins are packaged per portion and frozen at -20° C until needed at the Department of Human Biology in a freezer in room 3.247 , Universiteitssingel 50, that is only used to store food products. One batch of muffins is made for the entire study. We have chosen to supply the participants with muffins to mimic the texture of a real meal.

| **Ingredient** | **Brand** | **Bought at** |
| --- | --- | --- |
| flower | Euro shopper zelfrijzend bakmeel | Albert Heijn |
| sugar | AH suiker | Albert Heijn |
| egg | AH | Albert Heijn |
| butter | AH ongezouten roomboter | Albert Heijn |
| Low-fat milk | Campina | Albert Heijn |

# 5. METHODS

## 5.1 Study procedures

Subjects will come to the department twice for screening visits. During the first screening visit, the subjects will be weighed, blood pressure will be determined in four-fold (the first measurement will be discarded and the last three measurements will be averaged), body height will be determined, and a venous blood sample (5.5 mL) will be drawn for analysis of plasma lipids and glucose concentrations. During the second screening visit, body weight and blood pressure will be determined again and a second venous blood sample (3.5 mL) will be drawn for analysis of plasma lipids and glucose concentrations. Two screening visits are planned to reduce the within-subject variability in the parameters of interest.

On the day preceding the test day, subjects will be asked to avoid high fat foods, alcohol, caffeine and taking part in any strenuous activity/exercise (as these may influence lipid metabolism or other outcomes measures in the short-term). Subjects will be asked to fast overnight and will be instructed to avoid eating or drinking anything, except water, after 8 pm. Subjects will arrive at around 8.00 AM the following morning. Flow-mediated vasodilatation and pulse wave velocity measurements will be performed, a venous cannula (fixed needle) will be inserted and a fasting blood sample will be collected. During the FMD measurement, and ECG will be performed. Subjects will then consume the muffins and low-fat milk within 10 minutes. Further venous blood samples will be collected at regular intervals for up to 4 hours postprandially. During the 4 hours of the study, subjects will refrain from the consumption of any food or drink except water, which they will be asked to consume at regular intervals.

All venipunctures will be performed as much as possible by the same technician, at the same location, and at the same time of the day. Serum or plasma will be obtained by low-speed centrifugation and stored as appropriate. When possible, samples from one subject will be analyzed within one run at the end of the study under strict quality control. The blood-sampling scheme is given in Table 4.

The total amount of blood drawn will be 329 mL (5.5 mL during the two screening visits, 34.5 mL at days 0, 25, 56 and 81 and 90 mL at days 28 and 84) per person during the whole study. Therefore, subjects are not allowed to have donated blood 8 weeks prior to participation.

## 5.2 Study parameters/endpoints

Metabolic risk markers related to the metabolic syndrome: postprandial lipidemia, glycemia, insulinemia, and postprandial concentrations of factors reflecting low-grade systemic inflammation and endothelial function will be measured. Food intake, body weight and liver function will be measured as well. Based on new insights related to the original question, it may be decided to analyze the samples for other parameters related to the metabolic syndrome (metabolomics). Therefore, subjects will explicitly be asked in the informed consent form to consent (or not) with this approach. In addition, resveratrol concentration will be measured as well as safety parameters.

### 5.2.1 Metabolic risk markers

The main study endpoint will be fasting serum concentrations of apoA-I. Other metabolic risk markers that will be measured (see Table 3 for the exact time points of measuring) are:

- Fasting (total cholesterol, LDL-C, apoB, HDL-C and triacylglycerol) and postprandial lipemia (triglycerides)

- Fasting (hsCRP and IL-6) and postprandial markers for low-grade systemic inflammation (IL-6)

- Fasting and postprandial markers for endothelial function (sICAM-1, VCAM-1, MCP-1 and sE-selectin)

- Fasting (glucose and insulin) and postprandial glucose metabolism (glucose and insulin)

**5.2.2 Flow mediated vasodilatation**

Visualizing the response of the vessel wall to a regular daily stressor, which will be a fat rich muffin, is used to monitor characteristics of the vessel wall. This response will be measured at the end of the control and the intervention period, before and 4 hours after a fat load, by flow-mediated vasodilatation (FMD). FMD will be measured with established non-invasive ultrasound techniques that have been applied in earlier studies, in collaboration with the Department of Biophysics (Prof. A.P.G. Hoeks). For FMD, the diameter of the brachial artery will be measured before and after occlusion of the lower arm by a pneumatic tourniquet, with the subject in supine position. FMD is a non-invasive technique that makes use of ultrasound. During a FMD measurement, the subject lies in the supine position and the artery of the upper-arm will be visualized. After a person lay down for at least 15 minutes, and the artery is clearly visualized, the measurement starts. This means that we start a 3-minute baseline measurement, followed by 5 minutes inflation of a cuff that is positioned on the underarm. The cuff will be inflated at 50 mmHg above systolic pressure, which is measured on 3-minute intervals during the measurement. Inflation of the cuff will give a tingling feeling in the hand. The subject will be warned on this feeling, although there is no pain involved. We clearly ask the subjects to indicate it when the feel pain, as we will immediately deflate the cuff when this sensation is observed. The 5-minute inflation is followed by another 5-minute rest measurement. The total measurement is recorded on DVD, as FMD needs to be calculated afterwards.

### 5.2.3 Pulse wave velocity

Pulse wave velocity (PWV) measurements will be performed before and approximately 4 hours after a fat load to determine arterial distensibility and arterial stiffness. PWV measures pressure pulses by mechanotransducers, which are applied to the skin. One probe will be positioned at the site of the common carotid artery, whereas a second probe will be placed at the femoral artery site and a third one is placed at the site of the radial artery. At least 10 pulses should be recorded subsequently, after which PWV can be calculated from measurements of pulse transit time and the distance travelled by the pulse between the three recording sites. PWV is calculated automatically by the Complior device. PWV will be measured directly after FMD, to limit the (time) burden for the subjects.

### 5.2.4 Retinal images

Retinal images will be obtained to visualize the response of the microvasculature to the four weeks intake of resveratrol. Therefore, images of the retinal microvasculature will be made at the end of both intervention periods.

Images will be made at the policlinic outpatients’ department of ophthalmology at Maastricht University Medical Centre, in close consultation with the ophthalmologists and following standard procedures as applied during routine eye examinations. During this test, subjects are seated with the head resting on a chinrest, looking directly into the camera. The fundus camera will focus on and take a picture of the retina. The total procedure will take about 2 minutes. Apart from a widened pupil, which will be established by local tropicamide application (two drops in the eye), no physical side effects are expected during imaging. A Topcon TRC 50EX retinal camera combined with a Nikon D2Hs camera will be used to acquire the retina images. Afterwards, the images will be digitized and analyzed to calculate the AV-ratio with the appropriate software (Generalized Dual-Bootstrap Iterative Closest Point (GDB-ICP)) that is able to automatically initialize and individually match vascular landmarks. Next to this, the software will be used to measure retinal vessel diameter, i.e. mean of the four largest arteries or the four largest veins.

### 5.2.5 Clinical Chemistry

Samples from the start and end of each experimental period (see table 4) will be analyzed at the Department of Clinical Chemistry and the Department of Haematology of the University Hospital Maastricht, according to routine procedures.

- Blood chemistry: phosphorus, sodium, potassium, chloride, calcium, creatinine, blood urea nitrogen (BUN), protein, albumin, gammaGT, aspartate-aminotransferase (AST), alanine-aminotransferase (ALT), alkaline phosphatase (ALP), bilirubin
- Haematology: White cell count, red cell count, platelet count, hemoglobin, hematocrit, mean cell volume, and mean cell hemoglobin concentration, red cell distribution
- Blood coagulation: Prothrombin time (PT) and aPT

Table 3: Time points at which blood samples will be drawn and metabolic risk markers will be measured

| **Day** | **Screening**  **(2 visits)** | **Days 0 and 56** | **Days 25 and 81** | **Days 28 and 84** | | | | | | | | |
| --- | --- | --- | --- | --- | --- | --- | --- | --- | --- | --- | --- | --- |
| **Timepoint (T=)** |  |  |  | **0** | **15** | **30** | **45** | **60** | **90** | **120** | **180** | **240** |
| Serum |  |  |  |  |  |  |  |  |  |  |  |  |
| ApoA1 |  | X | X | X |  |  |  |  |  |  |  |  |
| ApoB |  | X | X | X |  |  |  |  |  |  |  |  |
| Total cholesterol | X | X | X | X |  |  |  |  |  |  |  |  |
| LDL-C | X | X | X | X |  |  |  |  |  |  |  |  |
| HDL-C | X | X | X | X |  |  |  |  |  |  |  |  |
| Insulin |  | X | X | X | X | X | X | X | X | X |  | X |
| Triglycerides |  | X | X | X |  |  |  | X |  | X | X | X |
| hsCRP |  | X | X | X |  |  |  |  |  |  |  |  |
| EDTA |  |  |  |  |  |  |  |  |  |  |  |  |
| IL-6 |  | X | X | X |  |  |  | X |  | X |  | X |
| sICAM-1 |  | X | X | X |  |  |  | X |  | X |  | X |
| VCAM-1 |  | X | X | X |  |  |  | X |  | X |  | X |
| MCP-1 |  | X | X | X |  |  |  | X |  | X |  | X |
| sE-Selectin |  | X | X | X |  |  |  | X |  | X |  | X |
| Haematology and Clinical Chemistry |  | X |  | X |  |  |  |  |  |  |  |  |
| Resveratrol |  | X | X | X |  |  |  |  |  |  |  |  |
| Heparin |  |  |  |  |  |  |  |  |  |  |  |  |
| Metabolomics |  | X |  | X |  |  |  |  |  |  |  | X |
| NaFl |  |  |  |  |  |  |  |  |  |  |  |  |
| Glucose | X | X | X | X | X | X | X | X | X | X |  | X |

### 5.2.6 Additional parameters

#### 5.2.6.1 Food intake

Habitual food intake will be measured at day 28 and day 84 using a validated food frequency questionnaire, which will immediately be checked by a dietician.

### 5.2.6.2 Body weight

Body weight without shoes and heavy clothing will be measured at each visit.

### 5.2.6.3 Resveratrol concentration

Plasma samples will be analyzed for resveratrol and its metabolites at days 0, 25, 28, and 56, 81, and 84 (see table 3).

Within 30 minutes after blood sampling, blood will be centrifuged for 15 minutes at ~2493 g at 4°Celsius. Immediately after centrifugation at least  1 ml of the cell free EDTA plasma supernatant will be transferred into “brown” 2 ml safe-lock polypropylene tubes (e.g. Eppendorf, order no. 0030 120.248). Samples will be put on ice, treated with nitrogen and stored as soon as possible at minus 80°Celsius until shipment to DSM.

For exposure control the analytics of resveratrol (original and metabolites) will be processed by LC/MS under *non*-GLP conditions. Liquid chromatography mass spectrometry (LC-MS) system is used for determination of “free” trans-resveratrol and  free  dihydroresveratrol (aglycone) and “total” trans-resveratrol and  total  dihydroresveratrol (aglycone + glucuronide conjugates) in biological fluids (serum  or  plasma). After addition of internal standard and liquid-liquid extraction (“free” analyte) or pre-digesting by β-glucuronidase followed by liquid-liquid-extraction (“total” analyte), the samples will be injected on a C18 column. Detection will be performed using MS in SIM mode.

Resveratrol samples will be sent deep frozen (on dry ice), together with a complete sample list to:

DSM Nutritional Products Ltd:

R&D Analytical Research Center (ARC)

Att.:  Sophia Vraka

 Building: 205, Room: 006

Wurmisweg 576, CH-4303 Kaiseraugst

Switzerland,

Phone: ++41 61 815  89 96

Fax: ++41 61 815 74 41

E-mail: [sample-reg-arc.kaiseraugst@dsm.com](mailto:sample-reg-arc.kaiseraugst@dsm.com)

The date of shipment will be arranged with representatives from DSM Nutritional Products, Dr. Iris Kunz and the registration desk ([sample-reg-arc.kaiseraugst@dsm.com](mailto:sample-reg-arc.kaiseraugst@dsm.com)) by e-mail. Samples will be sent on a Monday on dry ice, with a detailed sample list.

#### 5.2.6.4 Metabolomics

Metabolomics samples will be analyzed at days 0, 28, and 56, and 84 (see table 3). For this purpose, 3*1.5 mL heparin plasma is required (this includes one duplicate aliquot). 8-10 mL blood will therefore be collected in vacutainers (BD vacutainer heparin tubes, spray-coated with lithium heparin (Reference 367880)), and plasma will immediately be separated by centrifugation at 1500g for 15 minutes at 4°C, and then distributed into 2 light protected polypropylen or amber glas vials (one being the duplicate sample) for storage at or below -80^o^C.

Immediately after plasma preparation, “snap freeze” the samples: “Snap freezing” is done either by dipping the tubes into liquid nitrogen with forceps using protective equipment, or by snap-freezing them in an ethanol (95%)/dry ice mixture. Immediately after snap freezing, the tubes must be transfer to -80°C for storage. The samples will be shipped in batches to DSM for analysis. The duplicate samples will be stored at the study site (-80° C) until the end of the study.

Metabolomics samples will be sent deep frozen (on dry ice), together with a complete sample list to:

DSM Nutritional Products Ltd:

R&D Analytical Research Center (ARC)

Att.:  Sophia Vraka

 Building: 205, Room: 006

Wurmisweg 576, CH-4303 Kaiseraugst

Switzerland,

Phone: ++41 61 815  89 96

 Fax: ++41 61 815 74 41

E-mail: [sample-reg-arc.kaiseraugst@dsm.com](mailto:sample-reg-arc.kaiseraugst@dsm.com)

The date of shipment will be arranged with representatives from DSM Nutritional Products, Dr. Iris Kunz and the registration desk ([sample-reg-arc.kaiseraugst@dsm.com](mailto:sample-reg-arc.kaiseraugst@dsm.com)) and Ms Pascale Fuchs ([pascale.fuchs@dsm.com](mailto:pascale.fuchs@dsm.com)) by e-mail. Samples will be sent on a Monday on dry ice, with a detailed sample list.

#### 5.2.5.5 Compliance

Compliance to the study protocol will be checked by capsule count and by analyzing plasma samples on the intake of resveratrol and its metabolites at days 0, 25, 28, and 56, 81, and 84.

## 5.3 Withdrawal of individual subjects

Subjects can leave the study at any time for any reason if they wish to do so without any consequences. The investigator can decide to withdraw a subject from the study for urgent medical reasons or non-compliance.

## 5.4 Replacement of individual subjects after withdrawal

After withdrawal, subjects will not be replaced.

## 5.5 Follow-up of subjects withdrawn from treatment

After withdrawal, no follow-up of subjects will take place. In case of withdrawal due to medical complications, subjects will be referred to a general practitioner.

# 6. SAFETY REPORTING

## 6.1 Section 10 WMO event

In accordance to section 10, subsection 1, of the WMO, the investigator will inform the subjects and the reviewing accredited METC if anything occurs, on the basis of which it appears that the disadvantages of participation may be significantly greater than was foreseen in the research proposal. The study will be suspended pending further review by the accredited METC, except insofar as suspension would jeopardise the subjects’ health. The investigator will take care that all subjects are kept informed.

## 6.2 Adverse and serious adverse events

**Adverse Event (AE):** Any untoward medical occurrence in a subject involved in a biomedical research project administered an investigational product whether or not related to this product. An AE can therefore be any unfavourable and unintended sign (including an abnormal laboratory finding for example), subjective and objective symptom, or disease temporally associated with the use of a product, accidents, whether or not considered related to the product or study-related procedure and reported by the subject or observed by the investigator.

**Adverse Reaction (AR):** All noxious and unintended responses to a product related to any dose should be considered as adverse reactions.

**Serious Adverse Event (SAE):** Any AE that at any dose fulfils at least one of the following criteria:

- Is fatal (results in death) (*note*: death is an outcome, not an event)
- is life-threatening (*note*: the term “life-threatening” refers to an event in which the subject was at risk of death at the time of the event; it does not refer to an event which could hypothetically have caused death had it been more severe)
- requires inpatient hospitalization or prolongation of existing hospitalization (*note*: “inpatient hospitalization” refers to an unplanned, overnight hospitalization)
- results in persistent or significant disability/incapacity (*note:* the term means substantial disruption of one’s ability to conduct normal life function)
- is a congenital anomaly/birth defect (*note:* congenital anomaly/birth defect in offspring of subject taking the product regardless of time to diagnosis)
- is medically significant (*note*: Medical and scientific judgement should be exercised in deciding whether expedited reporting is appropriate in other situations, such as important medical events that may not be immediately life-threatening or result in death or hospitalisation but may jeopardise the subject or may require intervention to prevent one of the other outcomes listed in the definition above).

### 6.2.1 Suspected unexpected serious adverse reactions (SUSAR)

**Suspected Unexpected Serious Adverse Reaction (SUSAR):** All suspected adverse reactions related to a product (the tested product or active comparators) that are both unexpected and serious.

**Unexpected Adverse Reaction:** An adverse reaction, the nature, or severity or incidence of which is not consistent with the applicable product information (e.g. investigator’s brochure).

### 6.2.2 Adverse event assessment

**Expectedness:** An unexpected AE is an event of which the nature or severity is not consistent with the applicable product information.

**Causality Assessment:** The causality assessment of an AE to the investigational product will be rated as follows:

**- No (Not related):**

The temporal relationship of the clinical event to product administration makes a causal relationship unlikely, or other drugs, therapeutic interventions or underlying conditions provide a sufficient explanation for the observed event.

**- Yes (Related):**

The temporal relationship of the clinical event to product administration makes a causal relationship possible, and other drugs, therapeutic interventions or underlying conditions do not provide a sufficient explanation for the observed event.

**Severity / Intensity:** The severity / intensity of AEs will be graded on a three-point-scale:

- Mild or Grade 1: discomfort noted, but no disruption to normal daily activities.

- Moderate or Grade 2: discomfort sufficient to reduce or affect normal daily activities.

- Severe or Grade 3: Inability to work or perform normal daily activities.

**Outcome of event:** The outcome of an event will be classified as follows:

- Recovered

- Recovered with sequelae

- Ongoing

- Fatal

- Unknown / Lost to follow-up

### 6.2.3 Adverse event reporting

All AEs occurring during biomedical research projects involving human subjects are recorded in the CRF.

SAEs are reported and processed according to the applicable laws and regulatory requirements governing the conduct of biomedical research projects involving human subjects, i.e. all SAEs will be reported to the accredited METC that approved the protocol, according to the requirements of that METC.

Where appropriate, suspected unexpected serious adverse reactions (SUSARs) are reported and processed according to the applicable laws and regulatory requirements governing the conduct of biomedical research projects involving human subjects.

SAEs and SUSARs are additionally recorded on the SAE Form (**Appendix 3**) enclosed with the protocol. In case a pregnancy occurs it will be recorded on the pregnancy form (**Appendix 3**).

The filling-in of the SAE Form is detailed in the “Guidelines for completing the SAE Form” (**Appendix 4**) enclosed with the protocol.

The handling, processing and reporting of SAEs and SUSARs are described in detail in the study protocol and graphically presented in a flow-chart, enclosed with the study protocol (**Appendix 5**).

For SUSARs, study supplementation will be unblinded prior to reporting the case to the IRB/IEC and (if required) to the Health Authorities.

During or at the end of each study, an SAE reconciliation between data entered in the study database and the data entered in the pharmacovigilance database has to be performed if a pharmacovigilance database has been used. All discrepancies found during the reconciliation are documented in an SAE reconciliation report and corresponding queries are sent to the investigators for clarification.

### 6.2.4 Follow-up of adverse events

All adverse events will be followed until they have abated, or until a stable situation has been reached. Depending on the event, follow up may require additional tests or medical procedures as indicated, and/or referral to the general physician or a medical specialist.

# 7. STATISTICAL ANALYSIS

## 7.1 Descriptive statistics

Data will be presented as minimum, maximum, mean and standard deviation. Non-normally distributed parameters will be presented as minimum, maximum and medians.

## 7.2 Univariate analysis

For statistical analyses, the differences in effect of the placebo capsules and experimental capsules will be determined by comparing the changes between absolute concentrations as measured after the 4-week experimental period and the 4-week placebo period by using a paired T-test. Period and carry-over effects will be analyzed as described [21]. ANCOVA will be performed if baseline values are statistically different between the groups. If available, values of days 25 and 28 will be averaged to reduce within-subject variability.

Postprandial effects will be compared by using ANOVA with time, diet and subject number as factors. In all cases, a P-value <0.05 is considered to be statistically significant.

# 8. ETHICAL CONSIDERATIONS

## 8.1 Regulation statement

The study has to be approved by the Medical Ethical Committee of Maastricht University. The study will be conducted according to the Declaration of Helsinki and the Principles of GCP and in accordance with the Medical Research Involving Human Subjects Act (WMO).

## 8.2 Recruitment and consent

Subjects will be recruited among men and women in and near the vicinity of Maastricht by advertisements in local newspapers, the azM bulletin, the internet ([www.digi-prik.nl](http://www.digi-prik.nl)) and by posters in the university and hospital building. Besides this, subjects who have participated in earlier studies and who agreed to be approached for future studies will be contacted. Participation will be on a voluntary basis

Oral and written information on the purpose of the study will be given before the start of the study, after which written informed consent will be obtained. Informed consent should be signed before the start of the first screening visit. Subjects can consider participation for at least two days after the information is given. Subjects are free to approach an independent physician for further information and questions, and to withdraw at any stage of the study without further explanation.

## 8.3 Privacy

Personal data will be handled confidentially and stored in a password-protected file, to which only the investigators have access. Samples personal data will be coded and destroyed within five years. Only the investigators have access to the code.

## 8.4 Benefits and risks assessment, group relatedness

The subjects will record in diaries any signs of illness, medication used, deviations from the protocol and any experienced side effects such as headache, stomach complaints, nausea.

The interventions are safe and manufactured according to generally accepted procedures. Venipunctures can occasionally cause a local haematoma or bruise to occur. Some participants may report pain during venipuncture. Also, the FMD and PWV measurements are routine and are not expected to lead to side effects. For the retinal images, the eye pupil will be widened by Tropicamide, which is a commonly used technique at the Ophthalmology department. This pupil-widening will limit the subjects’ sight in the investigated eye. As this will last for a few hours, it is advised to wear sunglasses and not to drive a car.

Time investment of the subjects, as indicated in Table 4, is 16 hrs.

Table 4: Time investment of subjects

| Week | Blood sampling | Body weight, blood pressure | Diary / food frequency questionnaire | FMD measurements, postprandial test, retinal images | Total |
| --- | --- | --- | --- | --- | --- |
| Screening | | | | | |
| -2 | 10 min | 5 min |  |  | 15 min |
| -1 | 10 min | 5 min |  |  | 15 min |
| Intervention period | | | | | |
| 1 | 10 min | 5 min |  |  | 15 min |
| 4 | 2x10 min | 2x5 min | 10 min | 440 min | 460 min |
| 9 | 10 min | 5 min |  |  | 15 min |
| 12 | 2x10 min | 2x 5 min | 10 min | 440 min | 460 min |
| Total |  |  |  |  | 980 min |

## 8.5 Compensation for injury

The sponsor/investigator has a liability insurance which is in accordance with article 7, subsection 6 of the WMO. Maastricht University has an insurance, which is in accordance with the legal requirements in the Netherlands (Article 7 WMO and the Measure regarding Compulsory Insurance for Clinical Research in Humans of 23rd June 2003). This insurance provides cover for damage to research subjects through injury or death caused by the study.

€ 450.000,-- (i.e. four hundred and fifty thousand Euro) for death or injury for each subject who participates in the Research;

€ 3.500.000,-- (i.e. three million five hundred thousand Euro) for death or injury for all subjects who participate in the Research;

€ 5.000.000,-- (i.e. five million Euro) for the total damage incurred by the organisation for all damage disclosed by scientific research for the Sponsor as ‘verrichter’ in the meaning of said Act in each year of insurance coverage.

The insurance applies to the damage that becomes apparent during the study or within 4 years after the end of the study.

## 8.6 Incentives

Subjects will receive a compensation for the time invested in participating. This compensation will be €150 after complete participation or pro rata after withdrawal. No financial reward will be given for the screening visits, except for travelling costs. These costs will also be compensated during the study, on the basis of public transport, with a maximum of €0.19/km.

# 9. ADMINISTRATIVE ASPECTS AND PUBLICATION

## 9.1 Handling and storage of data and documents

Before the start of the screening, subjects will be assigned a random number that will not change during the study. This number is linked with the name, address, date of birth, and telephone number of the subject in a password-protected file. Except for the technicians and the dietician, only members of the project team can access this file. For all other purposes, the random number will be used for subject identification.

## 9.2 Amendments

Amendments are changes made to the research after a favourable opinion by the accredited METC has been given. All amendments will be notified to the METC that gave a favourable opinion.

## 9.3 End of study report

The investigator will notify the accredited METC of the end of the study within a period of 8 weeks. The end of the study is defined as the last patient’s last visit.

In case the study is ended prematurely, the investigator will notify the accredited METC, including the reasons for the premature termination.

Regarding the relatively short duration of the complete study we will not submit a summary of progress report.

Within one year after the end of the study, the investigator will submit a final study report with the results of the study, including any publications/abstracts of the study, to the accredited METC.

## 9.4 Public disclosure and publication policy

Publication policy is in agreement with the CCMO publication statement. Principal Investigator shall be free to publish and present the Results of the Study (“Publication”) provided that, until the end of the confidentiality period mentioned in article 20 of the Material Transfer Agreement with the producer of the product (DSM Nutritional Products), a copy of the proposed manuscript or abstract is given to DSM Nutritional Products for review at least 45 (forty-five) days prior to its submission for Publication. DSM Nutritional Products reserves the right to have deleted from such proposed manuscript or abstract any Confidential Information disclosed by DSM Nutritional Products to Principal Investigator under this Agreement which may be contained therein, provided, however, that Principal Investigator shall not be required to delete any basic information about the Material or its properties and functions that the Principal Investigator deems to be reasonably necessary to meaningfully convey the Results to their intended scientific audience. If DSM Nutritional Products does not notify Principal Investigator of any objections within 1 (one) month after the proposed manuscript or abstract has been given to DSM Nutritional Products, Principal Investigator shall be free to disclose the Publication. Results from this Study shall not be disclosed to any third party except pursuant to the publication procedure above.

Upon DSM Nutritional Products’ request or if so required by applicable Law, receipt of Material from DSM Nutritional Products will be acknowledged in each publication as “Material (or Material’s brandname if applicable) was provided by DSM Nutritional Products”.

# 10. REFERENCES

1. Nissen SE, Tsunoda T, Tuzcu EM, Schoenhagen P, Cooper CJ, Yasin M, et al. Effect of recombinant ApoA-I Milano on coronary atherosclerosis in patients with acute coronary syndromes: a randomized controlled trial. Jama. 2003 Nov 5;290(17):2292-300.

2. Sacks FM, Rudel LL, Connor A, Akeefe H, Kostner G, Baki T, et al. Selective delipidation of plasma HDL enhances reverse cholesterol transport in vivo. J Lipid Res. 2009 Jan 14.

3. Mooradian AD, Haas MJ, Wong NC. The effect of select nutrients on serum high-density lipoprotein cholesterol and apolipoprotein A-I levels. Endocr Rev. 2006 Feb;27(1):2-16.

4. Murphy AJ, Woollard KJ, Hoang A, Mukhamedova N, Stirzaker RA, McCormick SP, et al. High-density lipoprotein reduces the human monocyte inflammatory response. Arterioscler Thromb Vasc Biol. 2008 Nov;28(11):2071-7.

5. Rye KA, Barter PJ. Antiinflammatory actions of HDL: a new insight. Arterioscler Thromb Vasc Biol. 2008 Nov;28(11):1890-1.

6. Gotto AM, Jr. Low high-density lipoprotein cholesterol as a risk factor in coronary heart disease: a working group report. Circulation. 2001 May 1;103(17):2213-8.

7. Hertog MG, Feskens EJ, Hollman PC, Katan MB, Kromhout D. Dietary antioxidant flavonoids and risk of coronary heart disease: the Zutphen Elderly Study. Lancet. 1993 Oct 23;342(8878):1007-11.

8. Manach C, Mazur A, Scalbert A. Polyphenols and prevention of cardiovascular diseases. Curr Opin Lipidol. 2005 Feb;16(1):77-84.

9. Fan E, Zhang L, Jiang S, Bai Y. Beneficial effects of resveratrol on atherosclerosis. J Med Food. 2008 Dec;11(4):610-4.

10. Auger C, Teissedre PL, Gerain P, Lequeux N, Bornet A, Serisier S, et al. Dietary wine phenolics catechin, quercetin, and resveratrol efficiently protect hypercholesterolemic hamsters against aortic fatty streak accumulation. J Agric Food Chem. 2005 Mar 23;53(6):2015-21.

11. Do GM, Kwon EY, Kim HJ, Jeon SM, Ha TY, Park T, et al. Long-term effects of resveratrol supplementation on suppression of atherogenic lesion formation and cholesterol synthesis in apo E-deficient mice. Biochem Biophys Res Commun. 2008 Sep 12;374(1):55-9.

12. Erlund I, Koli R, Alfthan G, Marniemi J, Puukka P, Mustonen P, et al. Favorable effects of berry consumption on platelet function, blood pressure, and HDL cholesterol. Am J Clin Nutr. 2008 Feb;87(2):323-31.

13. Sacanella E, Vazquez-Agell M, Mena MP, Antunez E, Fernandez-Sola J, Nicolas JM, et al. Down-regulation of adhesion molecules and other inflammatory biomarkers after moderate wine consumption in healthy women: a randomized trial. Am J Clin Nutr. 2007 Nov;86(5):1463-9.

14. Stervbo U, Vang O, Bonnesen C. A review of the content of the putative chemopreventive phytoalexin resveratrol in red wine. Food Chemistry. 2006;101:449-57.

15. Boocock DJ, Faust GE, Patel KR, Schinas AM, Brown VA, Ducharme MP, et al. Phase I dose escalation pharmacokinetic study in healthy volunteers of resveratrol, a potential cancer chemopreventive agent. Cancer Epidemiol Biomarkers Prev. 2007 Jun;16(6):1246-52.

16. Duez H, Lefebvre B, Poulain P, Torra IP, Percevault F, Luc G, et al. Regulation of human apoA-I by gemfibrozil and fenofibrate through selective peroxisome proliferator-activated receptor alpha modulation. Arterioscler Thromb Vasc Biol. 2005 Mar;25(3):585-91.

17. Iannelli P, Zarrilli V, Varricchio E, Tramontano D, Mancini FP. The dietary antioxidant resveratrol affects redox changes of PPARalpha activity. Nutr Metab Cardiovasc Dis. 2007 May;17(4):247-56.

18. NCW W, inventor Use of resveratrol to regulate expression of apolipoprotein A-I.2006 Jul 6.

19. Jahagirdar R, Genest J, Hansen HC, Nicholls CD, Attwell S, McLure KG, et al. RVX-208: A Small molecule that increases ApoA-I, HDL-C, and cholesterol efflux. Atherosclerosis Supplements. 2008;9(1):2.

20. Spieker LE, Sudano I, Hurlimann D, Lerch PG, Lang MG, Binggeli C, et al. High-density lipoprotein restores endothelial function in hypercholesterolemic men. Circulation. 2002 Mar 26;105(12):1399-402.

21. Zern TL, Wood RJ, Greene C, West KL, Liu Y, Aggarwal D, et al. Grape polyphenols exert a cardioprotective effect in pre- and postmenopausal women by lowering plasma lipids and reducing oxidative stress. J Nutr. 2005 Aug;135(8):1911-7.

22. Zamora-Ros R, Urpi-Sarda M, Lamuela-Raventos RM, Estruch R, Vazquez-Agell M, Serrano-Martinez M, et al. Diagnostic performance of urinary resveratrol metabolites as a biomarker of moderate wine consumption. Clin Chem. 2006 Jul;52(7):1373-80.

23. Wenzel E, Somoza V. Metabolism and bioavailability of trans-resveratrol. Mol Nutr Food Res. 2005 May;49(5):472-81.
