# Supplementary material for: Resveratrol Does Not Influence Metabolic Risk Markers Related to Cardiovascular Health in Overweight and Slightly Obese Subjects: A Randomized, Placebo-Controlled Crossover Trial
Source: PLoS One. 2015 Mar 19;10(3):e0118393. doi: 10.1371/journal.pone.0118393 (PMC4366169; doi:10.1371/journal.pone.0118393)
Supplement: S1 Table — (DOCX) [file pone.0118393.s003.docx]

Supplemental Table S1

| Dietary intake during the placebo and resveratrol period, as estimated with food-frequency questionnaires | | | | | | | | | | |
| --- | --- | --- | --- | --- | --- | --- | --- | --- | --- | --- |
|  | **Placebo** | | | **Resveratrol** | | | **Difference** | | | ***P* value** |
| Energy (MJ/d) | 9.5 | ± | 2.9 | 9.9 | ± | 2.8 | 0.4 | ± | 2.0 | 0.180 |
| Energy (kcal/d)^2^ | 2273 | ± | 694 | 2373 | ± | 678 | 100 | ± | 482 | 0.190 |
| Carbohydrate (En%) | 45.9 | ± | 7.1 | 44.8 | ± | 7.7 | -1.1 | ± | 5.1 | 0.165 |
| Protein (En%) | 16.0 | ± | 3.2 | 15.5 | ± | 3.2 | -0.5 | ± | 2.6 | 0.205 |
| Total Fat (En%) | 36.4 | ± | 6.4 | 37.7 | ± | 6.8 | 1.3 | ± | 4.9 | 0.081 |
| SFAs (En%) | 12.0 | ± | 2.7 | 12.2 | ± | 2.6 | 0.2 | ± | 2.6 | 0.608 |
| MUFAs (En%)^2^ | 12.0 | ± | 2.6 | 12.9 | ± | 3.4 | 0.9 | ± | 2.3 | 0.022 |
| PUFAs (En%) | 9.1 | ± | 3.0 | 9.3 | ± | 3.5 | 0.2 | ± | 2.9 | 0.725 |
| Alcohol (En%)^2^ | 1.9 | ± | 3.1 | 2.2 | ± | 3.7 | 0.3 | ± | 1.6 | 0.478 |
| Dietary fiber (g/d) | 24.9 | ± | 7.7 | 24.6 | ± | 6.1 | -0.3 | ± | 5.2 | 0.716 |
| Cholesterol (mg/d) | 201 | ± | 71 | 202 | ± | 68 | -1 | ± | 60 | 0.884 |
| Values are means ± SD  ^1^ N = 45.  ^2^These parameters were tested by a Wilcoxon signed-rank test for non-normal distributed data. | | | | | | | | | | |
