# Supplementary material for: Resveratrol Does Not Influence Metabolic Risk Markers Related to Cardiovascular Health in Overweight and Slightly Obese Subjects: A Randomized, Placebo-Controlled Crossover Trial
Source: PLoS One. 2015 Mar 19;10(3):e0118393. doi: 10.1371/journal.pone.0118393 (PMC4366169; doi:10.1371/journal.pone.0118393)
Supplement: S2 Table — (DOCX) [file pone.0118393.s004.docx]

Supplemental Table S2

| Effect of 4-wk resveratrol intake on hematologic, hemostatic and general health parameters^1,2^ | | | | | | | | | | |  |
| --- | --- | --- | --- | --- | --- | --- | --- | --- | --- | --- | --- |
|  | **Placebo** | | | **Resveratrol** | | | **Difference** | | | ***P* value** | |
| Hemoglobin (mmol/L) (n=43)^3^ | 8.7 | ± | 0.6 | 8.6 | ± | 0.6 | -0.1 | ± | 0.3 | 0.199 | |
| Hematocryte (L/L) (n=43)^3^ | 0.42 | ± | 0.03 | 0.42 | ± | 0.03 | 0.00 | ± | 0.02 | 0.239 | |
| Erytrocytes (10^12^/L) (n=42)^3^ | 4.61 | ± | 0.29 | 4.58 | ± | 0.29 | -0.02 | ± | 0.18 | 0.568 | |
| MCV (fL) (n=43)^3^ | 91.3 | ± | 3.7 | 91.1 | ± | 3.5 | -0.1 | ± | 1.2 | 0.484 | |
| MCH (fmol) (n=44)^3^ | 1.84 | ± | 0.30 | 1.88 | ± | 0.10 | 0.04 | ± | 0.27 | 0.744 | |
| MCHC (mmol/L) (n=43) | 20.6 | ± | 0.5 | 20.6 | ± | 0.5 | 0.0 | ± | 0.4 | 0.821 | |
| Thrombocytes (10^9^/L) (n=43)^3^ | 237 | ± | 49 | 240 | ± | 49 | 1.4 | ± | 24 | 0.651 | |
| RDW (%) (n=39)^3^ | 13.5 | ± | 0.6 | 13.4 | ± | 0.6 | -0.1 | ± | 0.41 | 0.693 | |
| Leucocytes (10^9^/L) (n=43) | 5.5 | ± | 1.2 | 5.5 | ± | 1.4 | 0.0 | ± | 0.9 | 0.944 | |
| PT (s) (n=31)^3^ | 11.1 | ± | 1.4 | 11.5 | ± | 4.1 | 0.5 | ± | 3.0 | 0.904 | |
| aPTT (s) (n=32)^3^ | 27.8 | ± | 1.9 | 27.5 | ± | 1.8 | 0.1 | ± | 1.2 | 0.664 | |
| Sodium (mmol/L) | 142 | ± | 1.8 | 142 | ± | 1.4 | -0.1 | ± | 1.9 | 0.841 | |
| Potassium (mmol/L)^3^ | 4.31 | ± | 0.25 | 4.32 | ± | 0.29 | 0.01 | ± | 0.30 | 0.535 | |
| Calcium (mmol/L) | 2.31 | ± | 0.06 | 2.31 | ± | 0.08 | 0.00 | ± | 0.06 | 0.850 | |
| Phosphorus (mmol/L) | 1.05 | ± | 0.14 | 1.03 | ± | 0.13 | -0.02 | ± | 0.09 | 0.112 | |
| Chloride (mmol/L) | 104 | ± | 3.5 | 103 | ± | 3.4 | -0.2 | ± | 2.3 | 0.568 | |
| Ureum (mmol/L) | 5.0 | ± | 1.2 | 4.9 | ± | 1.4 | -0.1 | ± | 0.9 | 0.320 | |
| Creatinin (mmol/L) | 77.7 | ± | 11.7 | 77.3 | ± | 11.5 | -0.4 | ± | 5.7 | 0.640 | |
| ALP (U/L)^3^ | 81.8 | ± | 19.4 | 85.8 | ± | 22.6 | 4.0 | ± | 9.4 | 0.009 | |
| GGT (U/L)^3^ | 29.1 | ± | 18.9 | 28.7 | ± | 16.7 | -0.4 | ± | 12.7 | 0.955 | |
| ASAT (U/L)^3^ | 23.6 | ± | 11.0 | 24.8 | ± | 9.8 | 1.2 | ± | 5.8 | 0.308 | |
| ALAT (U/L)^3^ | 27.1 | ± | 25.6 | 25.4 | ± | 16.8 | -1.7 | ± | 17.4 | 0.567 | |
| Total bilirubin (μmol/L)^3^ | 10.1 | ± | 5.4 | 10.3 | ± | 6.1 | 0.2 | ± | 3.0 | 0.982 | |
| Total protein (g/L) | 70.8 | ± | 3.0 | 70.7 | ± | 3.6 | -0.1 | ± | 2.7 | 0.884 | |
| Albumin (g/L)^3^ | 43.1 | ± | 3.0 | 43.3 | ± | 3.1 | 0.1 | ± | 2.0 | 0.559 | |
| Values are means ± SD  ^1^N = 45, unless otherwise indicated.  ^2^ALAT, alanine aminotransferase; ALP, alkaline phosphatase; aPTT, activated partial thromboplastin time; AST, aspartate aminotransferase; GGT, γ-glutamyl transpeptidase; MCH, mean corpuscular hemoglobin; MCHC, mean corpuscular hemoglobin concentration; MCV, mean corpuscular volume; PT, prothrombin time; RDW, relative distribution weight  ^3^These parameters were tested by a Wilcoxon signed-rank test for non-normal distributed data. | | | | | | | | | | |  |
